# Epigenetic Reactivation of TNFRSF19 Suppresses Mitophagy and Sensitizes Triple‐Negative Breast Cancer to Doxorubicin

**DOI:** 10.1002/advs.77529

**Published:** 2026-08-31

**Authors:** Shiyang Liu, Chenguang Liu, Weihong Zheng, Xiaofei Tong, Zhengwei Gui, Zonghong Lu, Sun Ieong, Lin Zhang

**Affiliations:** ^1^ Department of Thyroid and Breast Surgery Tongji Hospital Tongji Medical College Huazhong University of Science and Technology Wuhan China; ^2^ Department of Thyroid and Breast Surgery Tianjin Key Laboratory of General Surgery Tianjin Cancer Institute of Integrative Traditional Chinese and Western Medicine Tianjin Union Medical Center, The First Affiliated Hospital of Nankai University Tianjin China

**Keywords:** DNA methylation, doxorubicin, mitophagy, TNFRSF19, triple‐negative breast cancer

## Abstract

Doxorubicin remains an important component of chemotherapy for triple‐negative breast cancer (TNBC), yet chemoresistance severely limits its clinical efficacy. Here, we identify Tumor necrosis factor receptor superfamily member 19 (TNFRSF19) as an epigenetically silenced gene that critically regulates doxorubicin response. Integrative analyses of The Cancer Genome Atlas (TCGA), Gene Expression Omnibus (GEO), and clinical cohorts reveal that high TNFRSF19 expression predicts superior pathological complete response and improved survival in doxorubicin‐treated TNBC patients. Mechanistically, TNFRSF19 binds the kinase domain of TGFBR1 via its intracellular domain, disrupting TGFBR1–SMAD3 complex formation and thereby inhibiting SMAD3 phosphorylation, nuclear translocation, and transcriptional activation of PTEN‐induced putative kinase 1 (PINK1). This suppresses PINK1/Parkin‐mediated mitophagy, contributing to mitochondrial dysfunction, reactive oxygen species (ROS) accumulation, and amplified DNA damage upon doxorubicin treatment. Notably, TNFRSF19 is downregulated in TNBC due to DNA hypermethylation, and decitabine restores its expression via promoter demethylation, thereby enhancing the therapeutic efficacy of doxorubicin in vitro and in vivo. Collectively, these findings establish TNFRSF19 as a critical epigenetic regulator of mitophagy, highlighting its potential as a predictive biomarker for doxorubicin response and a therapeutic target for sensitizing TNBC to doxorubicin.

## Graphical abstract

TNFRSF19 is an epigenetically silenced regulator of mitophagy in triple‐negative breast cancer. TNFRSF19 deficiency activates the TGFBR1–SMAD3–PINK1 axis to promote mitophagy and confer doxorubicin resistance, whereas decitabine‐mediated restoration of TNFRSF19 suppresses mitophagy and enhances doxorubicin sensitivity, revealing a targetable epigenetic–mitochondrial vulnerability in TNBC.

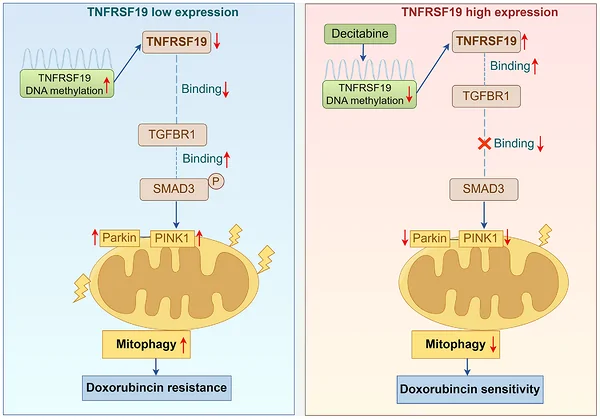

## 1 Introduction

Triple‐negative breast cancer (TNBC), characterized by the absence of estrogen receptor (ER), progesterone receptor (PR), and human epidermal growth factor receptor 2 (HER2), is one of the most aggressive breast cancer subtypes, with a high propensity for early recurrence and distant metastasis and a tendency to occur in younger women [1, 2, 3]. Therapeutic options for TNBC are limited to surgery, chemotherapy, and radiotherapy, as endocrine and HER2‐targeted therapies are ineffective due to receptor deficiencies [3, 4]. Anthracycline‐based chemotherapy, particularly doxorubicin, forms the backbone of first‐line TNBC treatment. Despite its efficacy in reducing 10‐year breast cancer mortality [2], doxorubicin's clinical efficacy is substantially limited by intrinsic or acquired chemoresistance [2, 5, 6, 7]. Overcoming doxorubicin resistance remains a critical challenge [8], necessitating novel strategies to enhance tumor chemosensitivity.

Mitophagy is a selective autophagic process that removes damaged mitochondria, thereby preventing cytosolic leakage of reactive oxygen species (ROS), mitochondrial DNA, and apoptotic activators to maintain cellular homeostasis and achieve mitochondrial quality control [9]. This process is primarily regulated by the PINK1‐Parkin pathway, in which mitochondrial membrane depolarization triggers PINK1 accumulation on the outer mitochondrial membrane, leading to Parkin recruitment and activation for ubiquitination of mitochondrial proteins and subsequent autophagic clearance [10]. Mitophagy is generally considered a defensive mechanism for cellular homeostasis [9]. By clearing dysfunctional mitochondria, mitophagy helps reduce oxidative stress and maintain mitochondrial function under metabolic stress, thereby promoting cancer cell survival and adaptation to unfavorable microenvironments [11, 12]. Notably, chemotherapeutic agents such as doxorubicin can induce protective mitophagy in response to mitochondrial stress and ROS accumulation, limiting oxidative damage and mitigating apoptosis, thereby facilitating drug resistance [13, 14]. Supporting this pro‐survival function, several studies have demonstrated that enhanced mitophagy facilitates breast cancer progression by maintaining mitochondrial homeostasis and facilitating adaptation to metabolic stress [15, 16, 17]. Conversely, mitophagy inhibition has been reported to increase doxorubicin sensitivity in colorectal cancer and enhance responsiveness to carboplatin and 5‐fluorouracil in esophageal squamous cell carcinoma [18, 19]. Specifically in TNBC, mitophagy inhibitors such as liensinine and Mdivi‐1 boost doxorubicin sensitivity by modulating apoptosis [20, 21]. Collectively, these findings position mitophagy inhibition as a promising strategy to reverse doxorubicin resistance in TNBC.

TNFRSF19, located at 13q12.12, is a member of the tumor necrosis factor receptor superfamily with diverse physiological roles, including regulating axonal growth, cell proliferation, differentiation, and cell death [22, 23, 24, 25, 26]. Critically, TNFRSF19 exhibits a context‐dependent dual role in cancer. It functions as an oncogene in nasopharyngeal carcinoma, melanoma, and glioblastoma [27, 28, 29]. Conversely, TNFRSF19 acts as a tumor suppressor in oral squamous cell carcinoma, liver, gastric, and lung cancers [30, 31, 32, 33]. Its potential as a therapeutic target is highlighted by the efficacy of CAR‐T cells incorporating TNFRSF19 domains against B‐cell malignancies [34]. Although our prior work established TNFRSF19 as a tumor suppressor in TNBC and noted its down‐regulated expression in tumor tissue [35], its role in modulating chemoresistance, particularly to doxorubicin, remains largely unexplored in TNBC.

In this study, we identify TNFRSF19 as an epigenetically silenced gene in TNBC that is associated with favorable pathological complete response and survival outcomes in doxorubicin‐treated patients. We uncover a previously unrecognized mechanism whereby TNFRSF19 disrupts TGFBR1–SMAD2/3 complex formation, suppressing SMAD3‐dependent PINK1 transcription, and inhibiting PINK1/Parkin‐mediated mitophagy. Furthermore, our data indicate that epigenetic reactivation of TNFRSF19 via DNA demethylation could enhance chemosensitivity. Collectively, these findings highlight TNFRSF19 reactivation as a potential strategy for improving doxorubicin response in TNBC.

## 2 Results

### 2.1 TNFRSF19 Serves as a Predictive Biomarker for Doxorubicin Response and Enhances the In Vivo Antitumor Efficacy of Doxorubicin in TNBC

**FIGURE 1:**
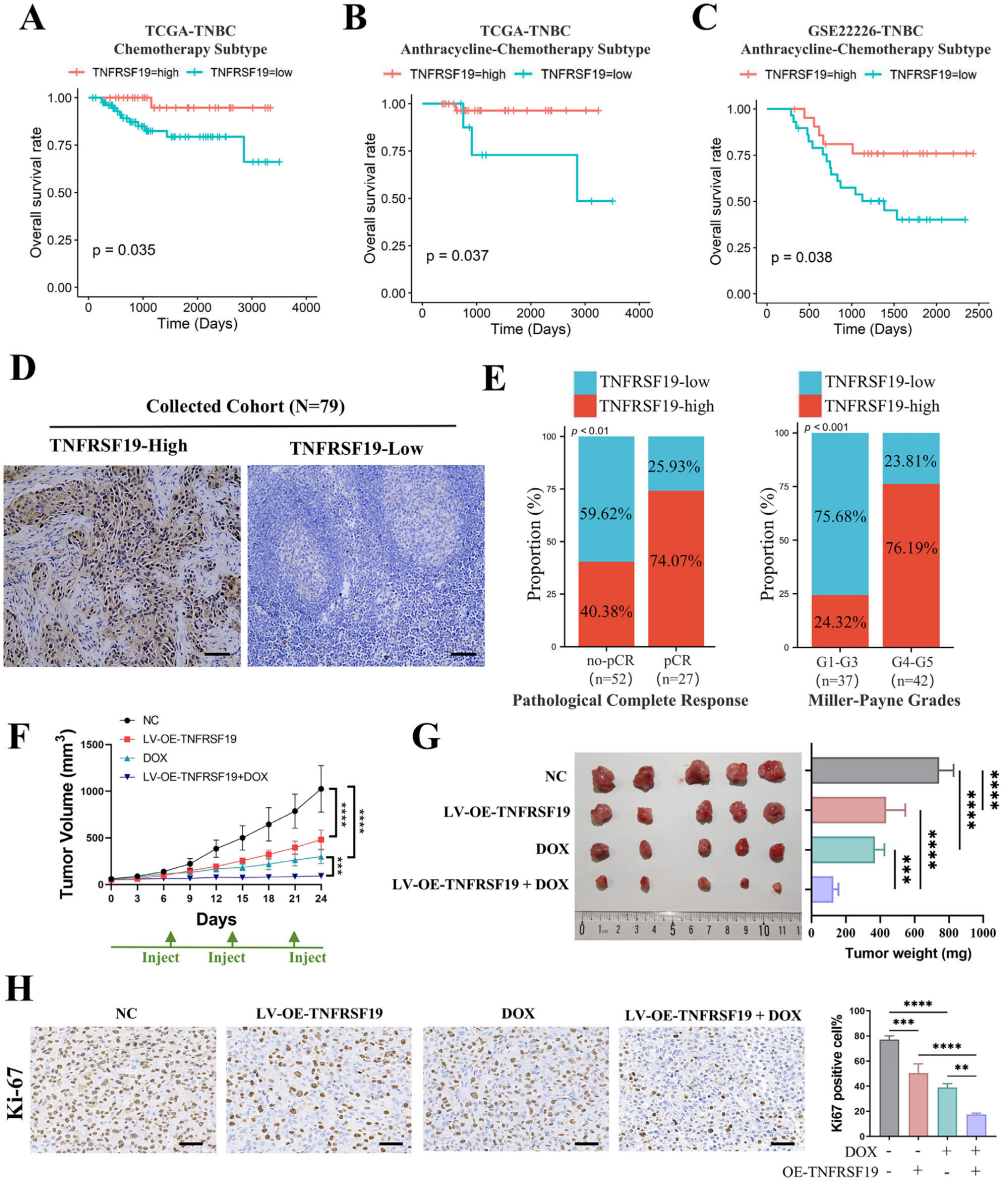
TNFRSF19 predicts favorable response to doxorubicin and enhances the in vivo antitumor efficacy of doxorubicin in TNBC. (A–C) Kaplan‐Meier survival curves show significantly improved overall survival in TNBC patients with high TNFRSF19 expression receiving chemotherapy in the TCGA chemotherapy cohort (*n* = 113), TCGA doxorubicin‐specific subgroup (*n* = 42), and GSE22226 doxorubicin‐treated subgroup (*n* = 51). (D) Representative immunohistochemistry (IHC) showing TNFRSF19 expression in collected TNBC tissues (High vs. low TNFRSF19 expression). Scale bars: 100 µm. (E) High TNFRSF19 expression is associated with a high pCR rate and a Miller‐Payne ‌grade G4–G5 rate. (F) Female Balb/c‐nu mice were randomly divided into four groups and subcutaneously injected with control or TNFRSF19‐overexpressing cells, followed by treatment with or without doxorubicin (*n* = 5 per group). Tumor growth curves were monitored until Day 24. (G) Representative images of subcutaneous tumors collected at the Day 24 endpoint and quantification of tumor weights (*n* = 5). (H) IHC for Ki‐67 in xenografts collected at the Day 24 endpoint (*n* = 5). TNFRSF19‐overexpressing tumors exhibited a lower percentage of Ki‐67‐positive cells. Scale bars: 50 µm. Data are presented as mean ± SD. P‐values were calculated using the chi‐square test (E), two‐way ANOVA (F), and one‐way ANOVA followed by Tukey's post hoc test (H). ^*^ *p* < 0.05, ^**^ *p* < 0.01, ^***^ *p* < 0.001, ^****^ *p* < 0.0001. pCR, pathological complete response.

Analysis of The Cancer Genome Atlas (TCGA) data revealed that elevated TNFRSF19 expression was significantly associated with improved overall survival in TNBC patients receiving chemotherapy (n = 113), particularly in the doxorubicin‐treated subgroup (*n* = 42) (Figure 1A,B). This association was further validated in the GSE22226 cohort of TNBC patients receiving doxorubicin‐based chemotherapy (*n* = 51) (Figure 1C). Consistently, analysis of our institutional cohort (*n* = 79) showed that low TNFRSF19 expression was associated with failure to achieve pathological complete response (pCR) and lower Miller‐Payne grades (G1–G3, <90% reduction in tumor cellularity), whereas high TNFRSF19 expression correlated with increased pCR rates and higher Miller‐Payne grades (G4–G5, ≥90% reduction in tumor cellularity) (Figure 1D,E and Table S1). To further determine whether TNFRSF19 modulates doxorubicin sensitivity in vivo, xenograft models were established. TNFRSF19 overexpression enhanced the antitumor effect of doxorubicin, resulting in a significantly greater reduction in tumor volume and tumor weight compared with either treatment alone (Figure 1F,G). Although TNFRSF19 overexpression alone exhibited a modest inhibitory effect on tumor growth, the greatest tumor suppression was observed when TNFRSF19 overexpression was combined with doxorubicin. Moreover, immunohistochemical analysis showed that Ki‐67 expression was reduced in TNFRSF19‐overexpressing tumors and was further decreased following combined treatment with doxorubicin (Figure 1H). Collectively, these findings identify TNFRSF19 as a predictive biomarker for doxorubicin response and demonstrate that TNFRSF19 enhances the therapeutic efficacy of doxorubicin in TNBC in vivo.

### 2.2 TNFRSF19 Sensitizes TNBC Cells to Doxorubicin In Vitro

**FIGURE 2:**
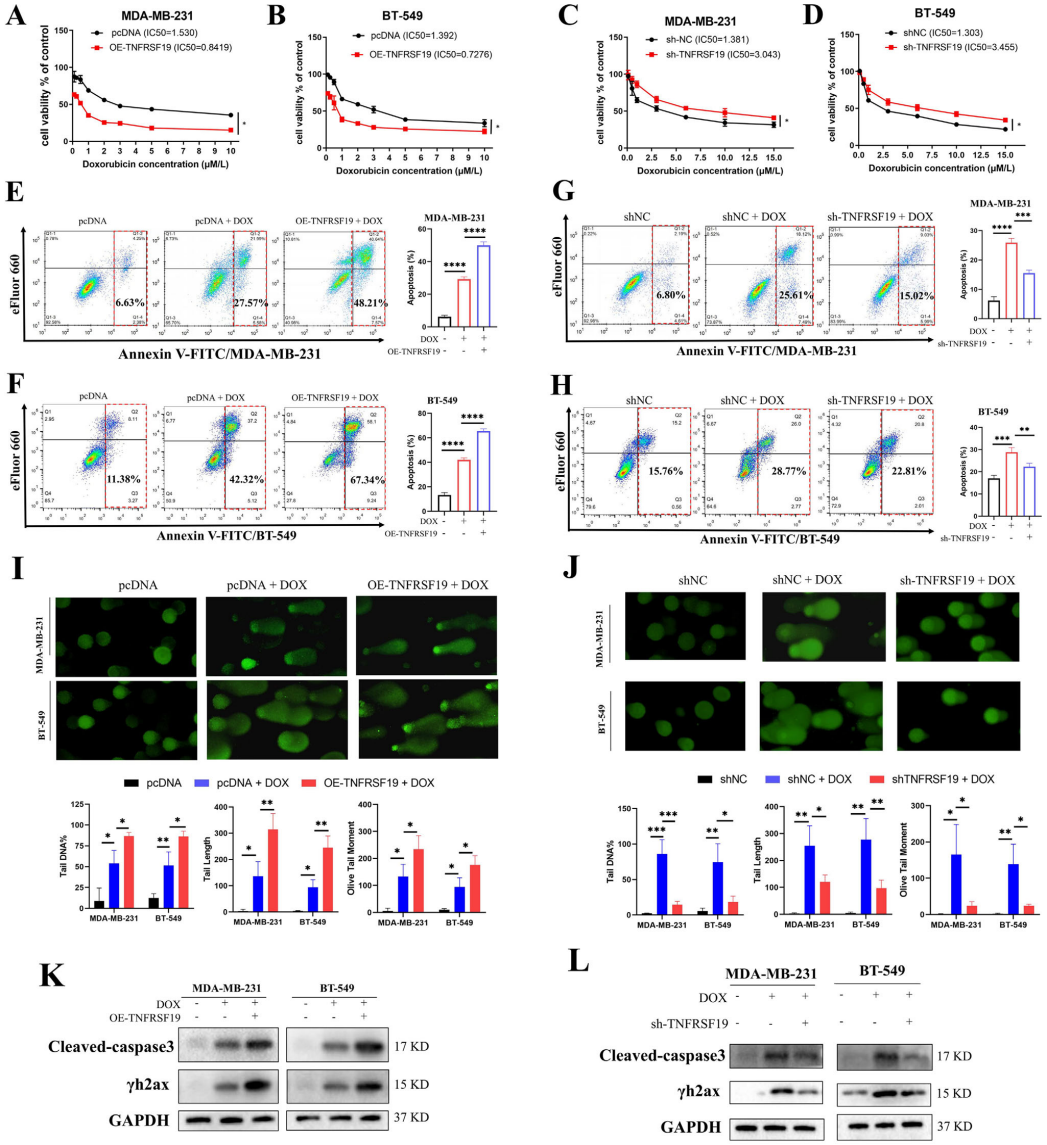
TNFRSF19 regulates doxorubicin sensitivity in TNBC cells. (A, B) CCK‐8 assays showing reduced viability and lower IC50 values in TNFRSF19‐overexpressing TNBC cells following doxorubicin (DOX) treatment. (C, D) TNFRSF19 knockdown attenuates DOX‐induced cytotoxicity and increases IC50 values. (E, F) Flow cytometry demonstrating increased apoptosis in TNFRSF19‐overexpressing cells. (G, H) TNFRSF19 knockdown reduces apoptosis in DOX‐treated cells. (I, J) Comet assays showing that TNFRSF19 overexpression enhances, whereas TNFRSF19 knockdown attenuates, DOX‐induced DNA damage (tail length, tail DNA%, Olive tail moment). (K, L) Western blot showing enhanced DOX‐induced γH2AX and cleaved caspase‐3 expression in TNFRSF19‐overexpressing cells and reduced expression upon TNFRSF19 knockdown. Data are presented as mean ± SD (*n* = 3). P‐values were calculated using two‐way ANOVA for cell viability and IC50 analyses (A–D), and one‐way ANOVA followed by Tukey's post hoc test for apoptosis and comet assay analyses (E–L). ^*^ *p* < 0.05, ^**^ *p* < 0.01, ^***^ *p* < 0.001, ^****^ *p* < 0.0001. IC50, half‐maximal inhibitory concentration; OE‐TNFRSF19, TNFRSF19 overexpression; pcDNA, empty vector control; sh‐TNFRSF19, TNFRSF19 knockdown; shNC, non‐targeting control.

Next, we evaluated the role of TNFRSF19 in doxorubicin sensitivity using in vitro assays. CCK‐8 assays under basal culture conditions showed that TNFRSF19 overexpression alone reduced cell viability, whereas TNFRSF19 knockdown had no significant effect under unstressed conditions (Figure S1). Upon doxorubicin treatment, TNFRSF19 overexpression significantly reduced cell viability and decreased IC50 values following doxorubicin (DOX) treatment (Figure 2A,B). In contrast, TNFRSF19 knockdown attenuated DOX‐induced cytotoxicity and increased IC50 values (Figure 2C,D). Consistently, flow cytometry analysis revealed that TNFRSF19 overexpression markedly increased apoptotic rates in DOX‐treated TNBC cells, whereas TNFRSF19 knockdown significantly reduced apoptosis (Figure 2E–H). To further assess DNA damage, comet assays showed that TNFRSF19 overexpression enhanced DOX‐induced DNA damage, as evidenced by increased tail length, tail DNA percentage, and Olive tail moment (Figure 2I). Conversely, TNFRSF19 knockdown reduced DNA damage under DOX treatment (Figure 2J). In agreement with these findings, western blot analysis demonstrated that TNFRSF19 overexpression enhanced DOX‐induced increases in γH2AX and cleaved caspase‐3 levels, whereas TNFRSF19 knockdown attenuated these effects (Figure 2K,L). Collectively, these results indicate that TNFRSF19 enhances doxorubicin sensitivity in TNBC cells by augmenting doxorubicin‐induced DNA damage and apoptosis.

### 2.3 TNFRSF19 Inhibits Mitophagy in TNBC Cells

**FIGURE 3:**
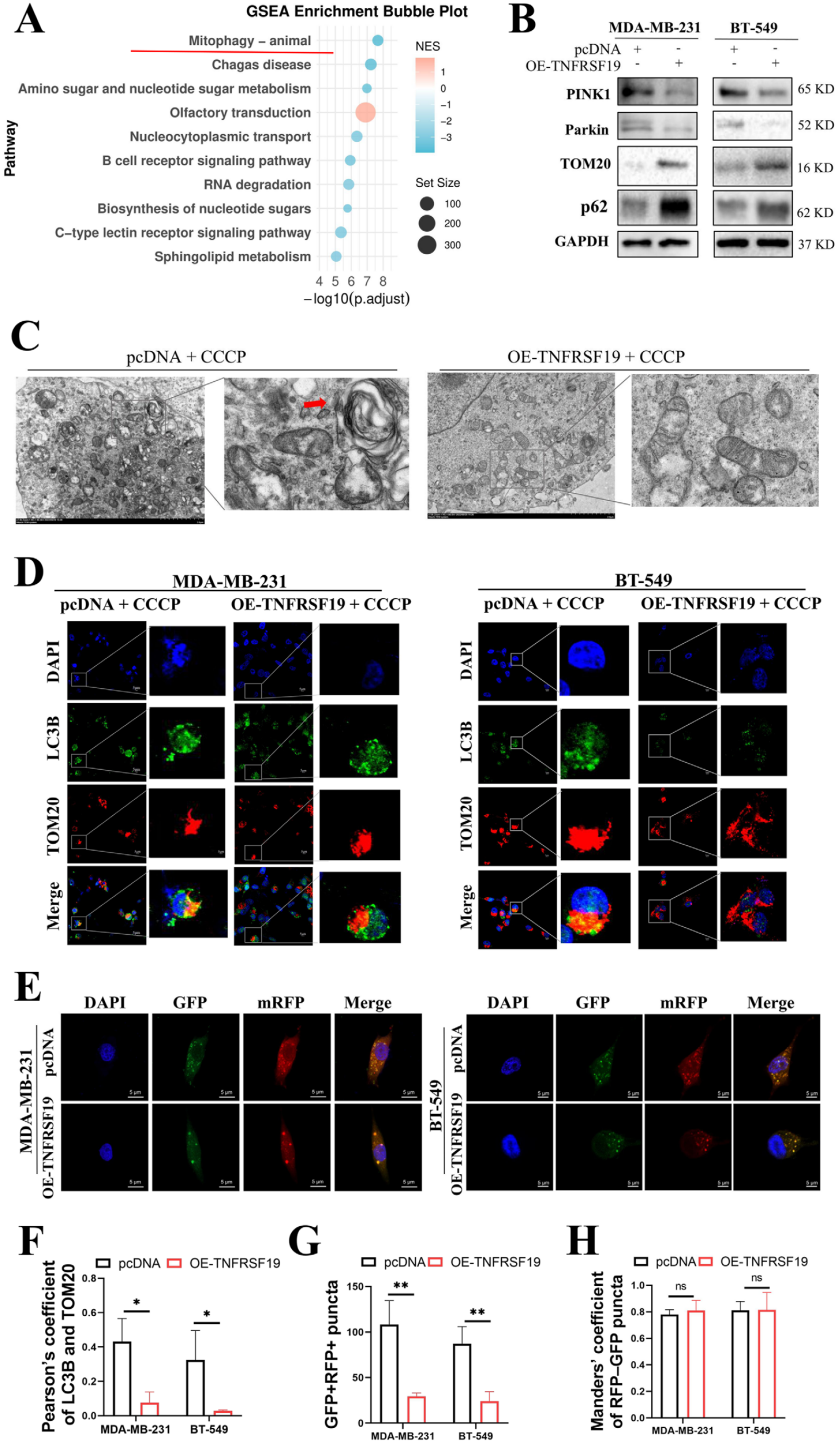
TNFRSF19 inhibits mitophagy in TNBC cells. ‌(A)‌ GSEA analysis of TCGA‐TNBC data showing mitophagy‐related pathway (Mitophagy‐animal) as the most negatively correlated gene set with TNFRSF19 expression. ‌(B)‌ Representative Western blots of PINK1, Parkin, TOM20, and p62 in TNFRSF19‐overexpressing TNBC cells. ‌(C)‌ Transmission electron microscopy showing mitochondrial morphology in TNBC cells after CCCP treatment. Red arrows indicate mitochondrial autophagy‐like structures in control cells (pcDNA + CCCP). Fewer mitochondrial autophagy‐like structures and more intact mitochondria were observed in TNFRSF19‐overexpressing cells (OE‐TNFRSF19 + CCCP). (D) Immunofluorescence of LC3B and TOM20 in TNFRSF19‐overexpressing cells. ‌(E)‌ Confocal images of TNFRSF19‐overexpressing TNBC cells transduced with the tandem mRFP–GFP–LC3B reporter, showing GFP^+^RFP^+^ puncta. (F) Quantification of LC3–TOMM20 co‐localization by Pearson's correlation coefficient (r) was calculated using ImageJ. (G) Quantification of GFP^+^RFP^+^ puncta per cell. (H) Quantification of RFP–GFP overlap by Manders’ coefficient. Data were presented as means ± SD; *n* = 3. Statistical analysis was performed using Student's t‐test (F–H). ^*^p < 0.05, ^**^p < 0.01, ^***^p < 0.001. ns, not significant. Scale bars: 5 µm. CCCP, carbonyl cyanide m‐chlorophenyl hydrazone.

To investigate the mechanisms underlying TNFRSF19‐mediated doxorubicin sensitization in TNBC, we performed gene set enrichment analysis (GSEA) to identify potential pathways associated with TNFRSF19 expression. GSEA of TCGA‐TNBC transcriptome data revealed that the mitophagy‐related pathway (Mitophagy‐animal) was the most negatively correlated gene set with TNFRSF19 expression (Figure 3A), suggesting a potential negative association between TNFRSF19 expression and mitophagy.

Western blotting confirmed that overexpression of TNFRSF19 in TNBC cells decreased PINK1 and Parkin levels while increasing TOM20 and p62 expression, whereas TNFRSF19 knockdown exerted the opposite effects (Figure 3B and Figure S2A), supporting TNFRSF19‐mediated inhibition of mitophagy. Transmission electron microscopy demonstrated fewer mitochondrial autophagy‐like structures and preservation of mitochondrial integrity in TNFRSF19‐overexpressing cells compared with controls (Figure 3C). Immunofluorescence analysis showed reduced colocalization of LC3B with TOM20 in TNFRSF19‐overexpressing cells (Figure 3D,F). Confocal imaging using the tandem mRFP‐GFP‐LC3B reporter further indicated that TNFRSF19 overexpression reduced the number of GFP^+^RFP^+^ puncta per cell without affecting the Manders’ coefficient, suggesting suppression of autophagosome formation rather than impaired maturation (Figure 3E,G–H). Collectively, these results establish TNFRSF19 as a negative regulator of mitophagy in TNBC cells.

Mitophagy flux analysis using Bafilomycin A1 (BafA1) confirmed that TNFRSF19 primarily suppresses mitophagy initiation rather than lysosomal degradation, as BafA1‐treated TNFRSF19‐overexpressing cells showed attenuated accumulation of PINK1, Parkin, and LC3‐II, accompanied by further increases in TOM20 and p62 compared with controls (Figure S3).

### 2.4 Mitophagy Suppression by TNFRSF19 Impairs Mitochondrial Quality Control and Enhances Chemosensitivity

**FIGURE 4:**
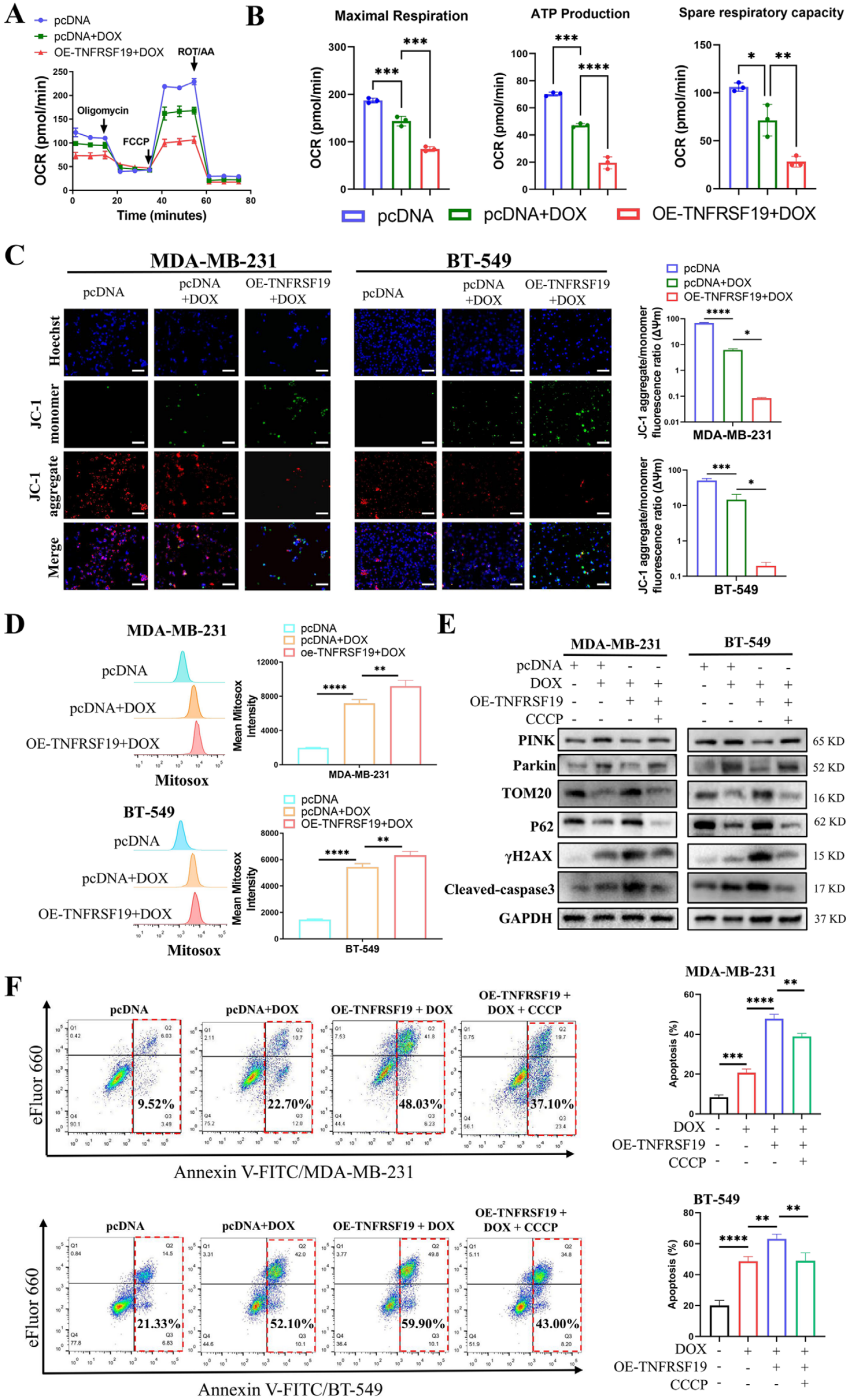
TNFRSF19 enhances doxorubicin sensitivity by suppressing mitophagy in TNBC cells. (A, B) Seahorse analysis shows that TNFRSF19 overexpression further decreases mitochondrial respiration in doxorubicin (DOX)‐treated cells, including reductions in maximal respiration and ATP production compared with DOX alone. (C) JC‐1 staining shows a pronounced loss of mitochondrial membrane potential (Δψm) in TNFRSF19‐overexpressing cells under DOX treatment, reflected by a significant decrease in the JC‐1 aggregates/monomers fluorescence ratio. Scale bars: 50 µm. (D) MitoSOX analysis shows elevated mitochondrial reactive oxygen species (ROS) in TNFRSF19‐overexpressing cells treated with DOX. (E) Representative Western blot showing reduced γH2AX and cleaved caspase‐3 levels in TNFRSF19‐overexpressing cells treated with doxorubicin and CCCP. ‌(F)‌ Flow cytometry analysis shows that the mitophagy activator CCCP reverses the increased apoptosis rates enhanced by TNFRSF19 overexpression under doxorubicin treatment. Data were presented as means ± SD; *n* = 3. P‐values were calculated using one‐way ANOVA followed by Tukey's post hoc test. ^*^p < 0.05, ^**^p < 0.01. ^***^p < 0.001, ^****^p < 0.0001. DOX, doxorubicin; CCCP, carbonyl cyanide m‐chlorophenyl hydrazone.

Building on the identification of TNFRSF19 as a negative regulator of mitophagy, we next investigated whether TNFRSF19‐mediated mitophagy suppression affects mitochondrial quality control and doxorubicin sensitivity. Seahorse analysis revealed that TNFRSF19 overexpression further decreased mitochondrial respiration under doxorubicin treatment, including reductions in maximal respiration, ATP production, and spare respiratory capacity compared with doxorubicin alone (Figure 4A,B). Consistently, JC‐1 staining demonstrated a greater loss of mitochondrial membrane potential (Δψm), while MitoSOX analysis demonstrated increased mitochondrial ROS accumulation in TNFRSF19‐overexpressing cells following doxorubicin exposure (Figure 4C,D). These findings indicate that TNFRSF19 overexpression exacerbates doxorubicin‐induced mitochondrial dysfunction and impairs mitochondrial quality control.

To determine whether mitophagy suppression contributes to TNFRSF19‐mediated chemosensitization, rescue experiments were performed using the mitophagy inducer CCCP. CCCP partially reversed TNFRSF19‐mediated enhancement of apoptosis and restored γH2AX and cleaved caspase‐3 levels in doxorubicin‐treated TNFRSF19‐overexpressing cells (Figure 4E,F). Conversely, the mitophagy inhibitor Mdivi‐1 restored the impaired DNA damage response and apoptosis caused by TNFRSF19 knockdown in doxorubicin‐treated cells (Figure S2B). These findings indicate that suppression of PINK1/Parkin‐mediated mitophagy is an important mechanism contributing to TNFRSF19‐mediated doxorubicin sensitization.

Importantly, we further investigated whether TNFRSF19‐mediated chemosensitization involved its previously reported MAPK/ER stress‐dependent paraptosis [35]. Pharmacological inhibition of MEK1/2 by U0126 failed to reverse TNFRSF19‐enhanced DNA damage and apoptosis following doxorubicin treatment (Figure S4). Moreover, neither U0126 nor the ER stress inhibitor 4‐PBA restored TNFRSF19‐induced suppression of mitophagy (Figure S5A,B). Furthermore, ALIX expression, a reported paraptosis‐associated marker, was not altered by doxorubicin treatment (Figure S5C). These findings suggest that TNFRSF19‐mediated regulation of mitophagy and chemosensitivity is distinct from its previously reported role in inducing paraptosis via MAPK/ER stress activation.

### 2.5 TNFRSF19 Interacts With TGFBR1 to Block TGFBR1/SMAD Complex Formation in TNBC Cells

**FIGURE 5:**
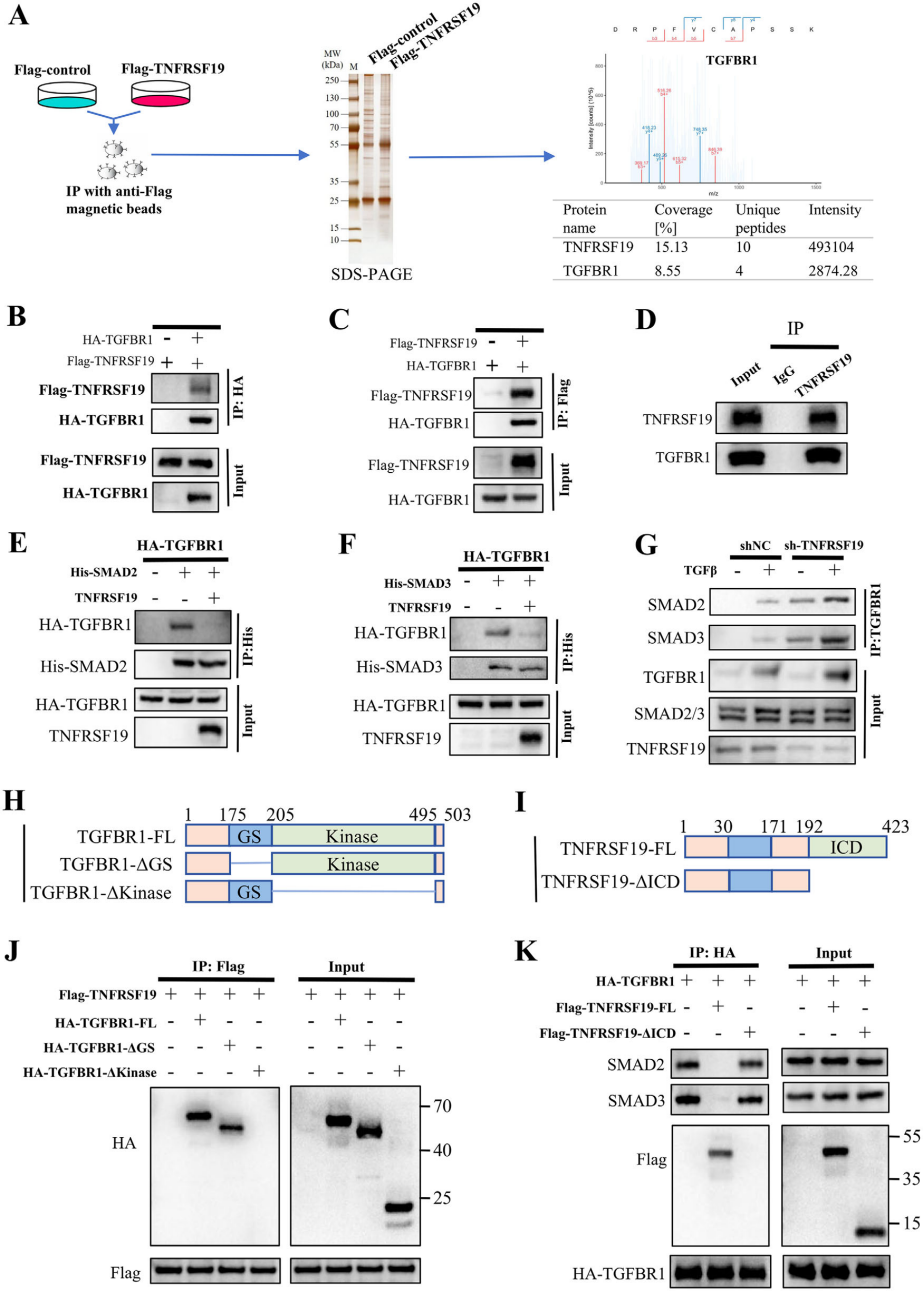
TNFRSF19 interacts with TGFBR1 to block TGFBR1/SMAD complex.‌ ‌(A)‌ Schematic workflow for Co‐IP/MS‐based profiling of TNFRSF19‐interacting proteins. ‌(B, C)‌ MDA‐MB‐231 cells were transfected with Flag‐TNFRSF19 or HA‐TGFBR1 plasmids, followed by immunoprecipitation with anti‐HA or anti‐Flag antibodies and immunoblot with anti‐Flag or anti‐HA antibodies, respectively. (D)‌ Detection of endogenous TNFRSF19‐TGFBR1 complexes in BT‐549 lysates using anti‐TNFRSF19 for immunoprecipitation and anti‐TGFBR1 for immunoblotting. ‌(E, F)‌ His‐tag pulldown assays were performed to evaluate TGFBR1‐SMAD2/3 interactions. HA‐TGFBR1 was co‐expressed with His‐SMAD2 or His‐SMAD3 in the presence or absence of TNFRSF19 overexpression, and the amount of co‐precipitated TGFBR1 was assessed. ‌(G)‌ Anti‐TGFBR1 immunoprecipitation assay evaluating endogenous TGFBR1‐SMAD2/3 interactions in wild‐type vs. TNFRSF19‐knockdown BT‐549 cells, with or without TGFβ stimulation (5 ng/mL). (H) Schematic representation of full‐length TGFBR1 and the truncated mutants (ΔGS and ΔKinase) used for domain‐mapping analysis. (I) Schematic representation of full‐length TNFRSF19 and the intracellular domain deletion mutant (ΔICD) used for interaction mapping. (J) Co‐IP analysis in MDA‐MB‐231 cells co‐transfected with Flag‐TNFRSF19 and HA‐TGFBR1 (FL, ΔGS, or ΔKinase). Deletion of the TGFBR1 kinase domain disrupted its interaction with TNFRSF19. (K) Co‐IP analysis in MDA‐MB‐231 cells co‐transfected with HA‐TGFBR1 and Flag‐TNFRSF19 (FL or ΔICD). Removal of the TNFRSF19 intracellular domain abolished its binding to TGFBR1. Notably, FL‐TNFRSF19 disrupted the TGFBR1–SMAD3 interaction, whereas the binding‐deficient ΔICD mutant did not. Co‐IP/MS, co‐immunoprecipitation coupled with mass spectrometry. FL, full‐length protein; ΔGS, GS domain deletion mutant of TGFBR1; ΔKinase, kinase domain deletion mutant of TGFBR1; ΔICD, intracellular domain deletion mutant of TNFRSF19.

To elucidate the molecular mechanisms by which TNFRSF19 modulates mitophagy, we identified transforming growth factor beta receptor 1 (TGFBR1) as an interacting protein of TNFRSF19 by co‐immunoprecipitation coupled with mass spectrometry (CO‐IP/MS) screening (Figure 5A). Further CO‐IP experiments confirmed the binding of TGFBR1 to TNFRSF19 in MDA‐MB‐231 and BT‐549 cells through both overexpression and endogenous validation (Figure 5B–D). Deng C et al. found that TNFRSF19 can competitively bind to TGFBR1 with SMAD2/3 in nasopharyngeal carcinoma, thereby blocking the TGF‐β pathway [27]. However, it is unclear whether this phenomenon exists in triple‐negative breast cancer. In MDA‐MB‐231 cells co‐expressing HA‐TGFBR1 and either His‐SMAD2 or His‐SMAD3 (R‐Smads), His‐tag immunoprecipitation assays revealed that the presence of Flag‐TNFRSF19 reduced the association of TGFBR1 with SMAD2 and SMAD3 (Figure 5E,F). Similarly, in BT‐549 cells with TNFRSF19 knockdown, CO‐IP experiments demonstrated that reduced expression of TNFRSF19 led to a significant enhancement in the protein complex formation between TGFBR1 and SMAD2/3 (Figure 5G). Building on previous studies showing that TNFRSF19's intracellular domain directly interacts with the kinase domain of TGFBR1 in nasopharyngeal carcinoma cells [27], we mapped the interaction domains in TNBC cells (MDA‐MB‐231) to determine whether this interaction is functionally required to disrupt TGFBR1–SMAD2/3 complex formation. The results showed that deletion of the TGFBR1 kinase domain (ΔKinase) disrupted its association with TNFRSF19, whereas the GS domain was dispensable (Figure 5H,J). These results indicate that the kinase domain of TGFBR1 is required for its interaction with TNFRSF19 in TNBC cells. Conversely, removal of the TNFRSF19 intracellular domain (ΔICD) abolished both its binding to TGFBR1 and its capacity to disrupt the TGFBR1–SMAD2/3 interaction (Figure 5I,K), demonstrating that the intracellular domain is required for TNFRSF19‐mediated inhibition of TGFBR1/SMAD signaling.

### 2.6 TNFRSF19 Inhibits the TGFBR1/SMAD Pathway and SMAD3‐Mediated PINK1 Transcription in TNBC Cells

**FIGURE 6:**
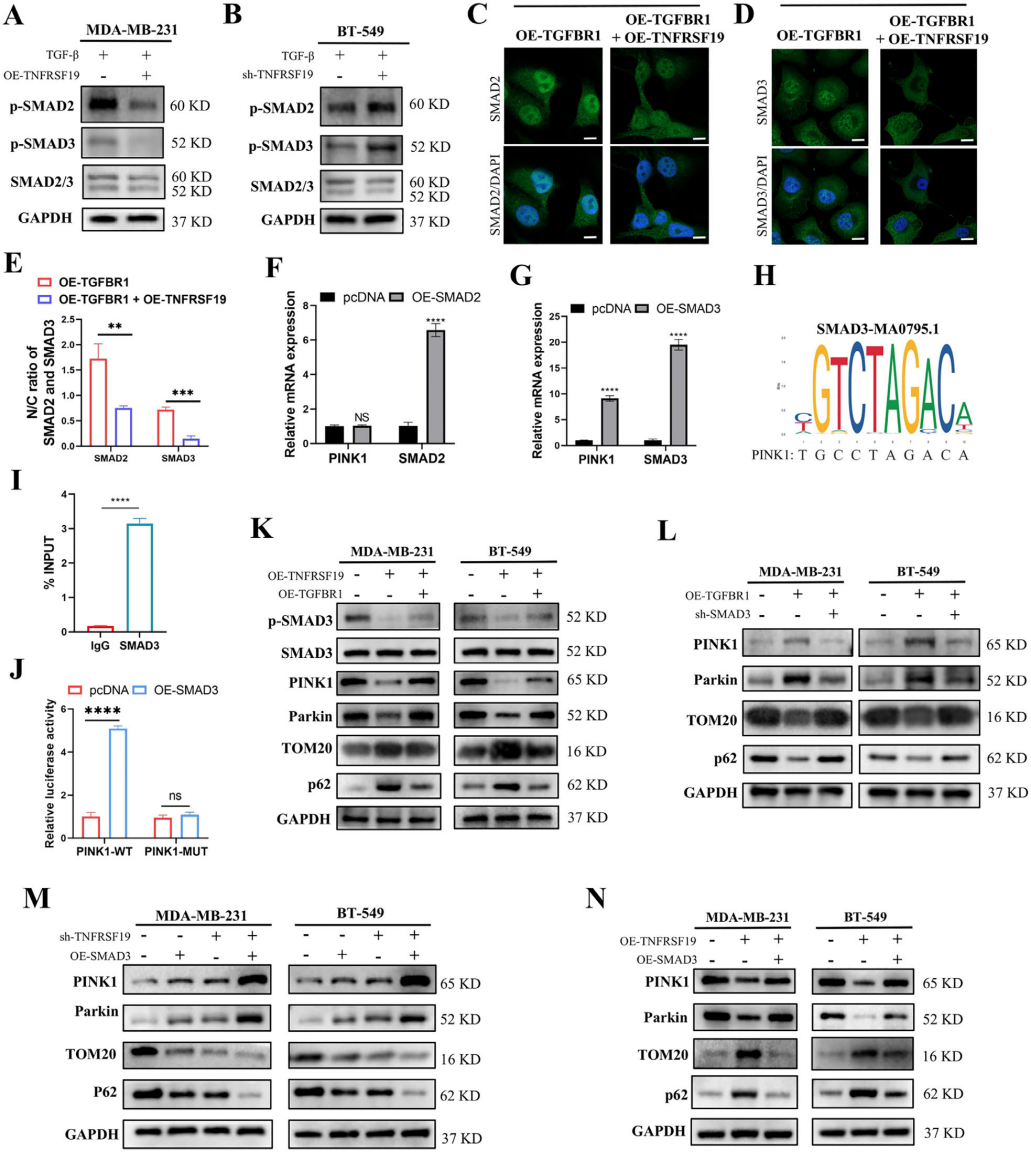
TNFRSF19 suppresses the TGFBR1‐SMAD3‐PINK1 axis to regulate mitophagy in TNBC cells.‌ ‌(A, B)‌ Western blot analysis demonstrating that TNFRSF19 overexpression markedly inhibits SMAD2/3 phosphorylation in TNBC cells. ‌(C, D)‌ Immunofluorescence showing that TNFRSF19 overexpression diminishes SMAD2 and SMAD3 nuclear accumulation. Scale bars: 5 µm. ‌(E) Nuclear/Cytoplasmic fluorescence intensity ratio (N/C ratio) of SMAD2 and SMAD3. ‌(F, G)‌ qRT‐PCR analysis in TNBC cells showing that SMAD3 overexpression leads to increased PINK1 mRNA levels, whereas SMAD2 overexpression has no effect. ‌(H)‌ Analysis of the JASPAR database revealing potential SMAD3 binding motifs in the PINK1 promoter. ‌(I)‌ ChIP‐qPCR analysis demonstrating the significant enrichment of SMAD3 at the predicted PINK1 promoter binding site in MDA‐MB‐231 cells. Results are expressed as a percentage of the total input DNA (% Input). ‌(J)‌ Assessment of luciferase activity driven by the wild‐type (WT) or mutated (MUT) PINK1 promoter in MDA‐MB‐231 cells upon SMAD3 overexpression, showing that SMAD3 enhances transcriptional activity of the WT promoter, whereas mutation abolishes this effect. (K) TNFRSF19 overexpression decreases SMAD3 phosphorylation, reduces PINK1 and Parkin, and increases TOM20 and p62, which are reversed by TGFBR1 restoration. (L) TGFBR1 overexpression elevates PINK1 and Parkin and reduces TOM20, whereas these effects are abolished by SMAD3 knockdown. (M) SMAD3 overexpression or TNFRSF19 knockdown increases PINK1 and Parkin and decreases TOM20 and p62; combined treatment further enhances these changes. (N) SMAD3 restoration reverses TNFRSF19‐mediated suppression of PINK1/Parkin expression and accumulation of TOM20 and p62 in TNFRSF19‐overexpressing cells. Data were presented as means ± SD; *n* = 3. Statistical analysis was performed using Student's t‐test (G, I). ^**^p < 0.01, ^***^p < 0.001, ^****^p < 0.0001. ns, not significant.

Based on the above findings, we hypothesize that TNFRSF19 may inactivate the TGF‐β/SMAD pathway in TNBC cells. Further GSEA analysis of TCGA‐TNBC data revealed an inverse correlation between the expression of TNFRSF19 and the TGFβ signaling pathway. Enriched pathways included: HALLMARK_TGF_BETA_SIGNALING (NES = −3.8708, P<0.01) and BIOCARTA_TGFB_PATHWAY (NES = −2.5088, P<0.01) (Figure S6A). Next, we investigated the downstream effects of the TGFBR1‐SMAD2/3 complex. Western blot analysis demonstrated that TNFRSF19 overexpression reduced SMAD2/3 phosphorylation, whereas TNFRSF19 knockdown increased SMAD2/3 phosphorylation in both MDA‐MB‐231 and BT‐549 cells (Figure 6A,B and Figure S7A,B). Moreover, quantitative fluorescence analysis revealed that TNFRSF19 overexpression markedly reduced the nuclear accumulation of SMAD2 and SMAD3, as evidenced by a decreased nuclear/cytoplasmic fluorescence intensity ratio (Figure 6C–E). Further Co‐IP assays revealed that overexpression of TNFRSF19 impeded the interaction between Smad2 and Smad4 (Co‐Smad), thereby preventing the formation of the active R‐Smad/Co‐Smad complex (Figure S6B). Conversely, in BT‐549 cells, TNFRSF19 knockdown markedly increased the formation of the Smad2‐Smad4 complex (Figure S6C). These findings indicate that TNFRSF19 interacts with TGFBR1 to block the TGFBR1/SMAD pathway.**‌**

Correlation analysis of TCGA‐TNBC datasets revealed a significant positive correlation between PINK1 expression and SMAD3 (R = 0.405, p = 0.001), whereas the association with SMAD2 was relatively weak (R = 0.199, p = 0.014) (Figure S8A,B). In TNBC cells, qRT‐PCR analysis showed that SMAD3 overexpression led to increased PINK1 mRNA levels, whereas SMAD2 overexpression had no effect (Figure 6F,G). Analysis of the JASPAR database (https://jaspar.elixir.no/) identified potential SMAD3 binding motifs in the PINK1 promoter (Figure 6H). To confirm direct binding, ChIP‐qPCR analysis was performed in MDA‐MB‐231 cells using an anti‐SMAD3 antibody. The result showed that SMAD3 was significantly enriched at the predicted binding site in the PINK1 promoter (Figure 6I), supporting a direct regulatory role for SMAD3 in PINK1 transcription. Furthermore, luciferase reporter assays demonstrated that SMAD3 overexpression markedly enhanced the transcriptional activity of the wild‐type PINK1 promoter, whereas mutation of the binding site completely abolished this effect, confirming the direct regulatory mechanism (Figure 6J).

To delineate the hierarchical regulation of the TNFRSF19–TGFBR1–SMAD3–PINK1 axis, we performed a series of rescue experiments. First, we examined whether TNFRSF19 suppresses mitophagy by antagonizing TGFBR1. Overexpression of TNFRSF19 markedly decreased SMAD3 phosphorylation and reduced PINK1 and Parkin expression, accompanied by accumulation of TOM20 and p62, whereas restoration of TGFBR1 expression in TNFRSF19‐overexpressing cells reversed these effects (Figure 6K), confirming that TNFRSF19 negatively regulates TGFBR1‐mediated SMAD3 phosphorylation and thereby inhibits mitophagy.

Next, we verified the requirement of SMAD3 in this signaling cascade. TGFBR1 overexpression alone elevated PINK1 and Parkin while reducing TOM20, and these effects were abolished by SMAD3 knockdown (Figure 6L), indicating that SMAD3 is required for TGFBR1‐mediated mitophagy. Consistently, in cells with TNFRSF19 depletion, SMAD3 overexpression further augmented the upregulation of PINK1 and Parkin and reduced TOM20 levels (Figure 6M), reinforcing the inverse functional relationship between TNFRSF19 and SMAD3. Moreover, restoration of SMAD3 expression in TNFRSF19‐overexpressing cells rescued the TNFRSF19‐induced reduction of PINK1 and Parkin and reversed the accumulation of TOM20 and p62 (Figure 6N), indicating restoration of mitophagy. In addition, SMAD3 overexpression alleviated the TNFRSF19‐mediated increase in γH2AX levels (Figure S9), demonstrating reduced doxorubicin‐induced DNA damage.

To further establish the functional relevance of the TNFRSF19 intracellular domain in mediating TNFRSF19‐dependent regulation of mitophagy, we compared the effects of full‐length TNFRSF19 and the intracellular domain deletion mutant (ΔICD). Unlike full‐length TNFRSF19, the ΔICD mutant failed to reduce PINK1/Parkin expression or induce TOM20 and p62 accumulation (Figure S10), indicating that the intracellular domain of TNFRSF19 is required for TNFRSF19‐mediated suppression of PINK1/Parkin‐mediated mitophagy. Collectively, these findings demonstrate that TNFRSF19 suppresses mitophagy and enhances doxorubicin‐induced DNA damage through inhibition of the TGFBR1–SMAD3–PINK1 signaling axis in TNBC cells.

### 2.7 TNFRSF19 is Regulated by DNA Methylation in TNBC

**FIGURE 7:**
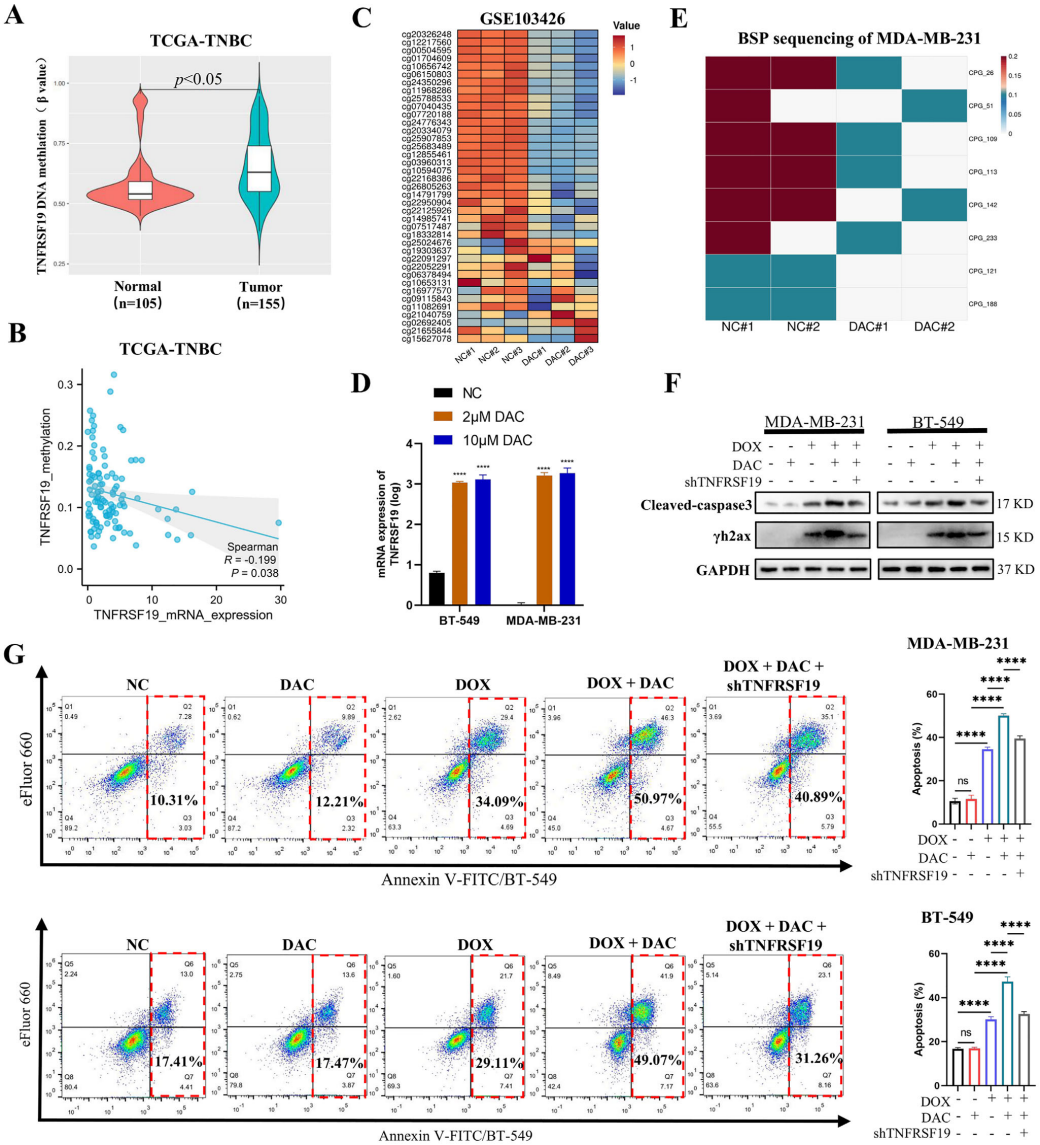
DNA methylation regulates TNFRSF19 to mediate chemosensitization in TNBC. (A) Data from TCGA‐TNBC reveal hypermethylation of TNFRSF19 in TNBC tumor tissue compared to normal tissue. (B) Data from TCGA‐TNBC reveal an inverse correlation between TNFRSF19 methylation levels and mRNA expression. (C) Data from GSE103426 reveal that decitabine reduces methylation at multiple loci of TNFRSF19 in MDA‐MB‐231 cells. (D) qRT‐PCR analysis showing increased TNFRSF19 mRNA expression following 2 or 10 µm decitabine treatment in MDA‐MB‐231 and BT‐549 cells. (E) BSP sequencing confirms TNFRSF19 promoter demethylation post‐decitabine treatment. (F) Representative western blots showing increased γ‐H2AX and cleaved caspase‐3 levels upon combined decitabine and doxorubicin treatment compared with doxorubicin alone, and these effects were abolished by TNFRSF19 knockdown. (G) Flow cytometry shows that TNFRSF19 depletion decreases the apoptosis rate induced by the decitabine/doxorubicin combination. Data were presented as means ± SD; *n* = 3. P‐values were calculated using Student's *t*‐test (A) or one‐way ANOVA followed by Tukey's post hoc test (E–G). ^****^p < 0.0001. ns, not significant. DOX, doxorubicin; DAC, decitabine.

Our previous study found that TNFRSF19 is downregulated in TNBC tissue [35]. Given its dual function as a tumor suppressor and enhancer of doxorubicin sensitivity, we further explored the mechanism underlying TNFRSF19 downregulation in TNBC. Analysis of methylation data from TCGA‐TNBC showed that TNFRSF19 was hypermethylated in TNBC compared with normal tissue (Figure 7A), and its mRNA levels were inversely correlated with promoter methylation (Figure 7B). Analysis of our institutional TNBC cohort (*n* = 79) revealed that higher TNFRSF19 promoter methylation was associated with reduced protein expression, with tumors exhibiting low TNFRSF19 protein expression showing significantly higher methylation levels than those with high TNFRSF19 protein expression (Figure S11A). Furthermore, patients without pathological complete response (no‐pCR) exhibited significantly higher TNFRSF19 methylation levels than those achieving pCR (Figure S11B). Moreover, patients with higher Miller–Payne grades (G4–G5) showed lower TNFRSF19 methylation compared with poor responders (G1–G3) (Figure S11C). These findings demonstrate that TNFRSF19 promoter hypermethylation is clinically associated with reduced TNFRSF19 expression and impaired response to anthracycline‐based therapy.

To validate whether DNA demethylation restores TNFRSF19 expression, we examined the effects of decitabine treatment. Analysis of the GSE103426 dataset revealed that decitabine reduced methylation at multiple TNFRSF19 loci in MDA‐MB‐231 cells (Figure 7C). The qPCR results showed that decitabine treatment at 2 and 10 µm significantly increased TNFRSF19 mRNA levels in both MDA‐MB‐231 and BT‐549 cells (Figure 7D). Based on these results, 2 µm decitabine was selected for subsequent promoter methylation and functional analyses. BSP sequencing confirmed demethylation of the TNFRSF19 promoter following 2 µm decitabine treatment (Figure 7E). We then assessed whether decitabine treatment could enhance the efficacy of doxorubicin through restoration of TNFRSF19 expression. Decitabine treatment alone did not induce significant DNA damage or apoptosis, as indicated by unchanged γH2AX, cleaved caspase‐3 levels, and apoptotic rates compared with the untreated control (Figure 7F,G). In contrast, combined decitabine and doxorubicin treatment markedly enhanced DNA damage and apoptosis compared with doxorubicin alone, and these effects were largely abrogated upon TNFRSF19 knockdown (Figure 7F,G). Collectively, these data indicate that decitabine‐mediated demethylation restores TNFRSF19 expression, thereby enhancing doxorubicin sensitivity in TNBC cells.

**FIGURE 8:**
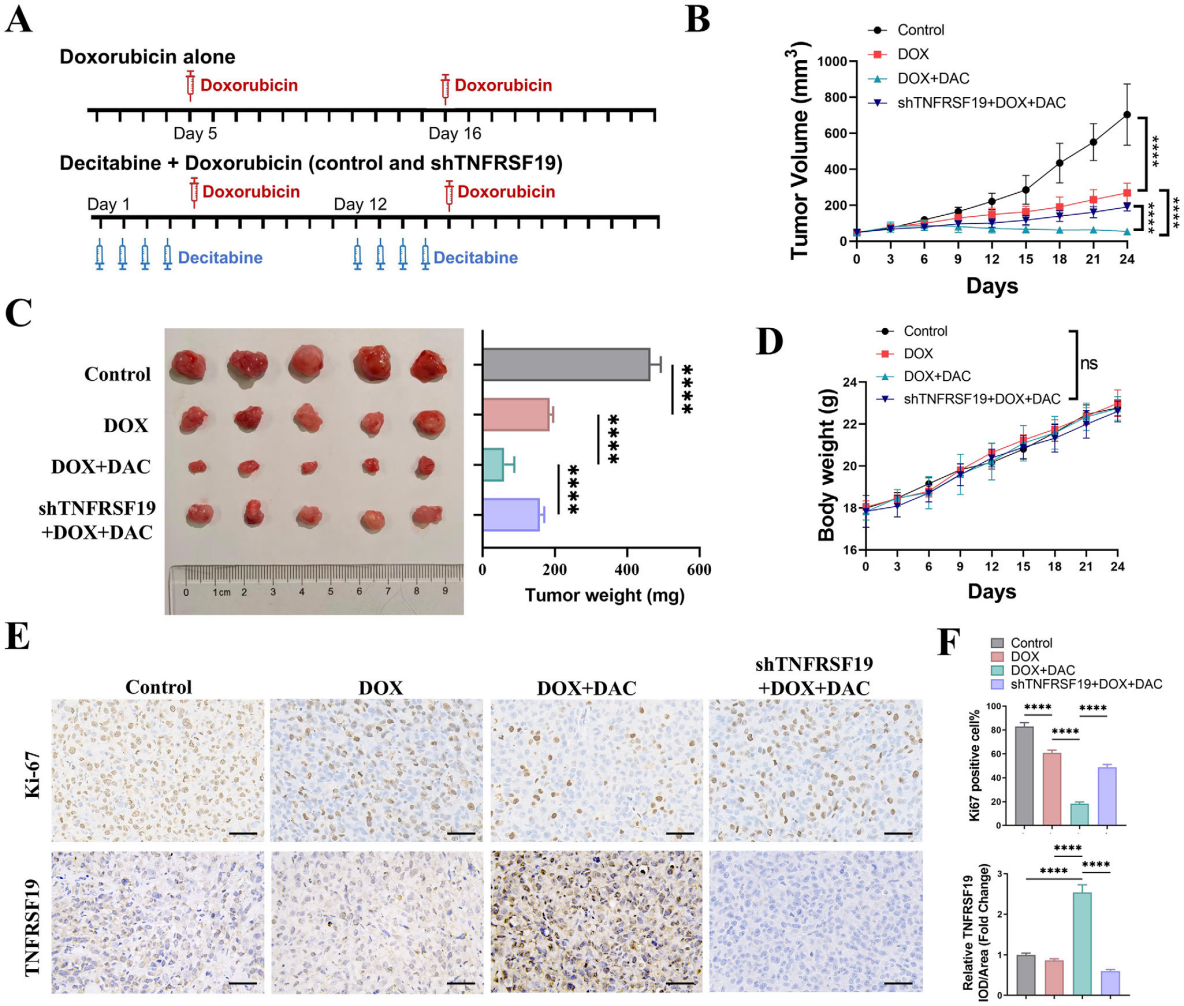
Decitabine enhances doxorubicin sensitivity in vivo. (A) Schematic illustration of the cyclic treatment regimen in the MDA‐MB‐231 xenograft model. Mice were divided into four groups (*n* = 5 per group): Control, doxorubicin alone (DOX), doxorubicin combined with decitabine (DOX + DAC), and TNFRSF19‐knockdown tumors treated with the combination of doxorubicin and decitabine (shTNFRSF19 + DOX + DAC). (B) Tumor growth curves showing that doxorubicin treatment inhibited tumor growth compared with the control group, whereas combined treatment with doxorubicin and decitabine further enhanced tumor suppression; this enhanced inhibitory effect was attenuated in TNFRSF19‐deficient tumors. (C) Representative images of excised tumors collected at the Day 24 and corresponding tumor weights (*n* = 5). (D) Body‐weight monitoring showing no significant differences among groups (*n* = 5). (E) Representative IHC staining for Ki‐67 and TNFRSF19 in xenograft tumors. Scale bars, 50 µm. (F) Quantification of Ki‐67–positive cells and relative TNFRSF19 expression (fold change of integrated optical density/area) (*n* = 5). The increase in TNFRSF19 expression and reduction in Ki‐67 induced by combined treatment with doxorubicin and decitabine were reversed by TNFRSF19 knockdown. Data are presented as mean ± SD. P‐values were calculated using two‐way ANOVA (B, D) and one‐way ANOVA followed by Tukey's post hoc test (C, F). ^***^p < 0.001, ^****^p < 0.0001. ns, not significant.

To verify these findings in vivo, xenograft models were established using MDA‐MB‐231 cells with or without TNFRSF19 knockdown. Mice received cyclic administration of decitabine followed by doxorubicin (Figure 8A). Compared with the control group, doxorubicin monotherapy significantly inhibited tumor growth. Moreover, the combination treatment with decitabine and doxorubicin further enhanced tumor suppression and reduced tumor weight compared with doxorubicin treatment alone (Figure 8B,C). Importantly, the enhanced antitumor effect induced by decitabine and doxorubicin combination treatment was markedly attenuated in TNFRSF19‐deficient tumors, supporting that TNFRSF19 reactivation contributes to the chemosensitizing effect of decitabine in vivo. No significant body‐weight changes were observed among groups, indicating good tolerability of the treatment regimen (Figure 8D). Immunohistochemical analysis further showed elevated TNFRSF19 and decreased Ki‐67 levels in tumors treated with the decitabine and doxorubicin combination, both of which were reversed by TNFRSF19 knockdown (Figure 8E,F). Collectively, these findings establish TNFRSF19 as an epigenetically silenced gene whose reactivation by decitabine enhances chemosensitivity to doxorubicin in TNBC, providing a mechanistic rationale for combining epigenetic therapy with anthracycline‐based chemotherapy.

## 3 Discussion

Doxorubicin remains the cornerstone of first‐line chemotherapy for TNBC, yet its clinical utility is significantly limited by chemoresistance [36]. Therefore, identifying new therapeutic targets to enhance the sensitivity of TNBC to doxorubicin is of great clinical significance. This study investigates the role of TNFRSF19 in modulating doxorubicin sensitivity in TNBC. Analysis of clinical data demonstrated that high TNFRSF19 expression correlates with improved prognosis in patients receiving doxorubicin‐based chemotherapy. Notably, elevated TNFRSF19 levels were associated with higher rates of pCR and favorable Miller‐Payne grades (G4–G5) following neoadjuvant chemotherapy. These clinical associations position TNFRSF19 as a promising predictive biomarker for chemotherapy response.

TNFRSF19, a member of the tumor necrosis factor receptor superfamily, has been identified as downregulated and serves as a tumor suppressor in various cancers, including oral squamous cell carcinoma, lung cancer, hepatocellular carcinoma, and gastric cancer [23, 25, 37, 38]. Additionally, TNFRSF19‐based CAR‐T cells show efficacy in targeting CD19‐positive B‐cell malignancies [39], highlighting its potential as an antitumor target. Our previous work demonstrated that TNFRSF19 suppresses TNBC progression through activation of MAPK‐dependent paraptosis [35]. However, its specific role in modulating chemotherapeutic response remained unexplored. To address this, we examined the effects of TNFRSF19 on doxorubicin sensitivity. Overexpression of TNFRSF19 enhances the inhibitory effects of doxorubicin on TNBC cell viability, increases apoptosis, amplifies DNA damage, and augments doxorubicin‐induced tumor growth inhibition in xenograft models. These results suggest that TNFRSF19 may improve the therapeutic efficacy of doxorubicin in TNBC.

Doxorubicin exerts its cytotoxic effects primarily through DNA intercalation and topoisomerase II inhibition, while simultaneously inducing apoptosis via reactive oxygen species (ROS) generation [40]. Chemoresistance is frequently driven by adaptive mitophagy, which clears ROS‐overloaded mitochondria and maintains pro‐survival thresholds, allowing tumor cells to survive under chemotherapeutic stress [14, 18, 38]. Inhibition of PINK1/Parkin‐mediated mitophagy enhances doxorubicin sensitivity, a phenomenon consistently observed in various cancer types that highlights mitophagy as a key mediator of chemoresistance [9, 15, 20, 21, 41]. We demonstrate that TNFRSF19 acts as an endogenous suppressor of PINK1/Parkin‐mediated mitophagy in TNBC cells. Mechanistic studies using Bafilomycin A1 turnover assays and tandem fluorescence reporters revealed that TNFRSF19 primarily inhibits the initiation of mitophagy, rather than impairing autophagosome maturation. As a consequence of mitophagy suppression, TNFRSF19 overexpression leads to the accumulation of dysfunctional mitochondria, resulting in elevated oxidative stress and increased susceptibility to apoptosis upon doxorubicin treatment. Importantly, CCCP‐mediated activation of mitophagy partially attenuated TNFRSF19‐induced chemosensitization, indicating that mitophagy suppression is an important contributing mechanism underlying TNFRSF19‐mediated effect. Collectively, these results reveal a previously unrecognized role of TNFRSF19 in suppressing pro‐survival mitophagy in TNBC, contributing to enhanced doxorubicin sensitivity.

To investigate the molecular mechanism by which TNFRSF19 regulates mitophagy, we conducted Co‐IP/MS analysis and identified an interaction between TNFRSF19 and TGFBR1. As the central receptor of TGF‐β signaling, TGFBR1 mediates SMAD2/3 phosphorylation, and subsequent nuclear translocation to orchestrate target gene transcription [42]. Previous study found that TGFBR1/SMAD signaling promotes breast cancer progression and doxorubicin resistance [43]. Besides, TGFBR1/SMAD signaling implicates in mitophagy regulation in ovarian cancer and hepatic stellate cells [44, 45]. However, TGFBR1/SMAD signaling's involvement in TNBC mitophagy regulation and potential crosstalk with TNFRSF19 are unknown. Our data demonstrate that TNFRSF19 competitively disrupts the TGFBR1‐SMAD2/3 interaction, thereby attenuating SMAD2/3 phosphorylation and impairing their nuclear accumulation.

Mechanistically, SMAD3, but not SMAD2, was identified as the key transcriptional activator of PINK1 in TNBC cells. In silico prediction and ChIP‐qPCR analysis confirmed that SMAD3 directly binds to the PINK1 promoter, while luciferase assays demonstrated that this binding is essential for SMAD3‐driven transcriptional activation of PINK1. These findings identify SMAD3 as a selective transcriptional regulator of mitophagy initiation in TNBC, consistent with previous reports demonstrating SMAD3‐mediated transcriptional activation of PINK1 in 293T cells [46]. Functional rescue assays further established the mechanistic link within the TNFRSF19–TGFBR1–SMAD3–PINK1 signaling axis. Overexpression of TGFBR1 reversed the inhibitory effects of TNFRSF19 on SMAD3 phosphorylation and mitophagy, supporting TGFBR1 as a critical downstream mediator of TNFRSF19 function. Moreover, restoring SMAD3 expression in TNFRSF19‐overexpressing cells reinstated PINK1/Parkin expression, further demonstrating that the TGFBR1–SMAD3 pathway is a key downstream mediator of TNFRSF19‐mediated mitophagy suppression. Collectively, these findings delineate a previously unrecognized signaling mechanism in which TNFRSF19 interferes with TGFBR1‐mediated SMAD3 activation, thereby repressing PINK1 transcription and mitophagy initiation in TNBC. This suppression of mitophagy compromises mitochondrial quality control and enhances doxorubicin sensitivity, positioning TNFRSF19 as a pivotal regulator of mitochondrial homeostasis, and chemotherapeutic response in triple‐negative breast cancer.

DNA hypermethylation at CpG‐rich promoters is a well‐established mechanism for silencing tumor suppressor genes by obstructing transcription factor binding, a process frequently deregulated in cancer [47, 48, 49]. Given the emerging evidence of DNA hypermethylation‐mediated gene suppression, we explored whether TNFRSF19 downregulation in TNBC involves epigenetic regulation. Clinical and TCGA data indicate that higher TNFRSF19 promoter methylation is associated with reduced expression and poor pathological response to chemotherapy, suggesting that epigenetic silencing contributes to TNFRSF19 deficiency in TNBC. Decitabine is a clinically approved hypomethylating agent for myelodysplastic syndromes and acute myeloid leukemia [50]. Previous studies have demonstrated that decitabine‐based epigenetic priming strategies can enhance responses to conventional chemotherapy and immunotherapy in solid tumors, including ovarian cancer, bladder cancer, and sarcomas [51, 52, 53]. By increasing tumor sensitivity to cytotoxic agents, decitabine‐based epigenetic combination strategies may provide an opportunity to optimize treatment regimens and potentially reduce dose‐related toxicities [54, 55]. In this study, we demonstrate that decitabine induces TNFRSF19 promoter demethylation and restores TNFRSF19 expression, thereby enhancing doxorubicin‐induced DNA damage and apoptosis. Importantly, this chemosensitizing effect was substantially attenuated by TNFRSF19 knockdown, indicating that TNFRSF19 reactivation represents an important mechanism underlying decitabine‐mediated sensitization.

This study has several important limitations. First, the clinical cohort analyzed in this study was relatively small and derived from a single center, and follow‐up survival data were not yet available, which may limit the statistical power and the ability to assess long‐term prognostic significance. Second, although our xenograft models confirmed the in vivo effects of TNFRSF19 on doxorubicin sensitivity, patient‐derived xenograft (PDX) models are required to further validate these findings in a more clinically relevant setting. In addition, while our data support a role for PINK1/Parkin‐mediated mitophagy in TNFRSF19‐induced chemosensitization, the incomplete rescue by CCCP suggests that additional pathways may also contribute to this process and warrant further investigation. Furthermore, because decitabine is a global DNA methyltransferase inhibitor, additional epigenetically regulated genes may also contribute to its chemosensitizing effects, although TNFRSF19 appears to be an important mediator of this process.

## 4 Conclusion

Collectively, we identify TNFRSF19 as a methylation‐silenced gene that enhances doxorubicin sensitivity in TNBC by inhibiting the TGFBR1–SMAD3–PINK1 axis and suppressing mitophagy. Therapeutic strategies aimed at reactivating TNFRSF19 in combination with doxorubicin may represent a promising strategy to improve responsiveness to anthracycline‐based therapy in TNBC.

## 5 Experimental Section

### 5.1 Data collection

TNBC cases were identified from The Cancer Genome Atlas (TCGA, https://cancergenome.nih.gov/) and cBioPortal for Cancer Genomics. TNBC was defined as tumors negative for estrogen receptor, progesterone receptor, and HER2 by immunohistochemistry (IHC), or negative HER2_neu_in_situ_hybrid_outcome_type status. A total of 155 TNBC cases were included, among which 113 patients had received chemotherapy, and 42 were annotated as having received doxorubicin/adriamycin‐based regimens (TCGA anthracycline subgroup). Public microarray data (GSE22226) comprising 51 TNBC patients treated with doxorubicin and cyclophosphamide‐based chemotherapy.

In addition, 79 TNBC patients with complete clinicopathological data, who received uniform anthracycline‐based neoadjuvant chemotherapy at our institution, were included. All patients in the institutional cohort were treated with a standard AC–T regimen consisting of doxorubicin (60 mg/m^2^) and cyclophosphamide every 3 weeks for four cycles, followed by paclitaxel. Pathological complete response (pCR) was defined as ypT0 ypN0, indicating the complete absence of residual invasive and in situ carcinoma in the breast and the absence of residual tumor in axillary lymph nodes. The study protocol was approved by the institutional ethics committee, and written informed consent was obtained from all participants.

For survival analyses in the TCGA‐TNBC and GSE22226 cohorts, patients were dichotomized into high‐ and low‐TNFRSF19 expression groups using an optimal cutoff value determined by maximally selected rank statistics. In the institutional cohort, TNFRSF19 expression was evaluated by IHC, and patients were stratified into high‐ and low‐expression groups using the median IHC score as the cutoff value.

### 5.2 GSEA Analysis

Based on TCGA‐TNBC cohort analyses, patients were stratified into TNFRSF19‐high and ‐low expression groups using median mRNA expression as the threshold. Differential gene expression between groups was analyzed via the limma R package. Subsequent Gene Set Enrichment Analysis (GSEA) implemented by clusterProfiler identified significantly enriched pathways, with significance defined as |NES| >1, nominal p < 0.05, and FDR < 0.25.

### 5.3 Cell Culture, Plasmid Transfection, and Lentiviral Infection

MDA‐MB‐231 (RRID: CVCL_0062) and BT‐549 (RRID: CVCL_1092) were obtained from the Shanghai Institute of Cell Biology within the last 3 years, and authenticated via short tandem repeat profiling. MDA‐MB‐231 and BT‐549 were cultured in DMEM and RPMI‐1640 media (Hyclone, USA), respectively, supplemented with 10% fetal bovine serum (Gibco, USA). Human TNFRSF19 overexpression plasmid (TSINGK, China), human TNFRSF19‐targeting shRNA, and TNFRSF19 lentiviral infection were conducted according to our previous study [35].

### 5.4 Immunohistochemistry

Paraffin‐embedded tissue sections were processed through deparaffinization, rehydration, and inhibition of endogenous peroxidase activity. They were then incubated with primary antibodies overnight at 4°C. Afterward, the sections were exposed to a biotinylated secondary antibody and washed. The immunostaining results were visualized using the Aperio ImageScope system by Leica Biosystems. Two pathologists independently assessed the immunohistochemistry score, which was determined by combining the staining intensity level (ranging from 0 for no staining to 3 for intense staining) and the proportion of stained cells (ranging from 0 for none to 4 for over 75%). Based on this scoring system, samples were classified into groups with either high or low expression of TNFRSF19, using the median score as the threshold.

### 5.5 Comet Assay

Cell suspension (5 × 10^3^ cells) was mixed with low‐melting agarose (37°C) and plated on CometSlides (biotechne, USA). After solidification (4°C, 30 min), slides were lysed overnight (4°C), then treated with alkaline unwinding solution (300 mM NaOH, 1 mM EDTA, pH > 13). Electrophoresis was conducted in prechilled alkaline buffer (300 mM NaOH, 1 mM EDTA, pH > 13). Post‐electrophoresis, slides were washed, ethanol‐fixed, and stained with SYBR Green (Thermofisher, USA). DNA damage was quantified via CASP software using tail length, % tail DNA, and Olive tail moment.

### 5.6 Flow Cytometry

Apoptosis was assessed using an Annexin V‐FITC Kit (Invitrogen, USA) per manufacturer's instructions. During DOX treatment, dead cells were detected with eFluor 660 (Invitrogen) to avoid fluorescence interference. After filtering cells through a 300‐mesh nylon mesh, apoptosis rates were quantified by flow cytometry (BD Biosciences, USA).

### 5.7 Quantitative Real‐Time Polymerase Chain Reaction (qPCR)

Total RNA was extracted using Trizol reagent (Invitrogen, China) and reverse transcribed with a kit (Novazan, China). qPCR was conducted on a system (Bio‐Rad, USA) using SYBR Green mix (Novazan, China) and primers, with gene expression normalized to GAPDH. Primer sequences: TNFRSF19 forward 5'‐GACCTCAGCTCCACGAATATG‐3', reverse 5'‐CACCCCACAACCAAGAGTCG‐3'; GAPDH forward 5'‐GGAGCGAGATCCCTCCAAAAT‐3', reverse 5'‐GGCTGTTGTCATACTTCTCATGG‐3'; PINK1 forward 5'‐CAAGAGAGGTCCCAAGCAAC‐3', reverse 5'‐GGCAGCACATCAGGGTAGTC‐3'.

### 5.8 Western Blotting

Protein samples were extracted using RIPA buffer (Servicebio) supplemented with protease inhibitors. Following quantification, proteins underwent electrophoretic separation and subsequent transfer to PVDF membranes. Primary antibody incubation (4°C, 12 h) was performed according to Table S2, followed by 1 h room temperature incubation with HRP‐conjugated secondary antibodies (1:5000). Protein signals were detected using Thermo Fisher's high‐sensitivity ECL system. All experiments were independently repeated at least three times with consistent results, and representative images are shown.

### 5.9 Immunofluorescence

The cells were fixed in 4% paraformaldehyde, followed by 0.05% Triton X‐100 in chilled PBS. After that, the cells were incubated with a primary antibody overnight at 4°C. This was followed by the application of a Cy3‐conjugated or FITC‐conjugated secondary antibody (Servicebio, China). The IF samples were then examined using a confocal fluorescence microscope (Olympus, Japan). For quantitative analysis of nuclear/cytoplasmic localization (SMAD2/3) or co‐localization (LC3‐TOM20), at least 30 cells per group were analyzed from three independent experiments using ImageJ software. Pearson's correlation coefficients were calculated to assess co‐localization.

### 5.10 Electron Microscopy Observations

For ultrastructural analysis, collected cells underwent primary fixation in 2.5% glutaraldehyde with subsequent secondary fixation using 1% osmium tetroxide. Progressive ethanol dehydration preceded resin embedding. Semithin sections (<50 nm) were obtained before dual staining with uranyl salts and lead citrate. Final observation was performed on a transmission electron microscope system (FEI, USA).

### 5.11 Adenovirus mRFP‐GFP‐LC3B Transfection

Seeded MDA‐MB‐231 and BT‐549 cells were transfected with GFP‐RFP‐LC3B adenovirus (TSINGK, China) at optimal MOI. The medium was replaced with PBS and then with complete medium prior to imaging by confocal microscopy (GFP/RFP channels). For quantification of autophagosomes and autolysosomes, at least 30 cells per group were analyzed from three independent experiments using ImageJ software. GFP^+^RFP^+^ (autophagosomes) and RFP^+^ only (autolysosomes) puncta were counted, and Manders’ or Pearson's coefficients were calculated to assess co‐localization.

### 5.12 Immunoprecipitation and Mass Spectrometry

To identify TNFRSF19‐interacting proteins, cellular lysates were prepared in immunoprecipitation buffer, clarified by ultracentrifugation, with a 50 µL aliquot reserved as input control. The remaining supernatant underwent co‐immunoprecipitation overnight at 4°C using specific antibodies coupled to Protein A/G magnetic beads, followed by three washes and denaturation in loading buffer via boiling. Samples were separated by SDS‐PAGE with silver staining, and distinct bands analyzed by LC‐MS using a Q Exactive mass spectrometer, with data processed in MaxQuant against the UniProt human database.

### 5.13 Xenograft Assay

All animal experiments were approved by the Animal Care and Use Committee of our institution (approval number: TJH‐2023040005). Female 6‐week‐old BALB/c nude mice were injected subcutaneously with 2 × 10^6^ MDA‐MB‐231 cells stably transduced with a control vector (NC) or TNFRSF19‐overexpression lentivirus (LV‐OE‐TNFRSF19). For the doxorubicin‐response experiment, mice were randomly assigned into four groups (*n* = 5 per group): NC, LV‐OE‐TNFRSF19, DOX, and LV‐OE‐TNFRSF19 combined with DOX. Mice received intraperitoneal (i.p.) administration of doxorubicin (3 mg/kg) once weekly according to the experimental schedule. Tumor volume and body weight were measured every 3 days.

For the decitabine‐doxorubicin combination experiment, mice were injected with control or TNFRSF19‐knockdown cells, and received decitabine (0.5 mg/kg, i.p.) prior to doxorubicin (3 mg/kg, i.p.) following a two‐cycle regimen based on a previously established protocol with modifications [56]. Tumor volume and body weight were monitored every 3 days. At the study endpoints, mice were euthanized, and tumors were excised, weighed, and fixed in 4% paraformaldehyde in accordance with ARRIVE guidelines [57].

### 5.14 Chromatin Immunoprecipitation (ChIP) and qPCR

Chromatin immunoprecipitation was carried out using the Simple ChIP Plus Enzymatic Chromatin IP Kit (Thermofisher, USA) following the supplier's guidelines. Briefly, MDA‐MB‐231 cells were cross‐linked with 1% formaldehyde, lysed, and chromatin was fragmented to 200–1000 bp by sonication. Immunoprecipitation was carried out using anti‐SMAD3 antibody or normal rabbit IgG as a control. DNA was purified following reversal of cross‐links and analyzed by quantitative PCR using primers targeting the SMAD3‐binding region of the PINK1 promoter, identified based on JASPAR prediction, and previously reported conserved SMAD3‐responsive elements [46] (Forward: 5′‐AAAAGATGGGAGATGTTATGGG‐3′; Reverse: 5′‐AGCCATGTGGAACTGTAAGT‐3′). Data were normalized to input DNA and expressed as a percentage of total input.

### 5.15 Luciferase Reporter Assay

The wild‐type (WT) or mutant (MUT) PINK1 promoter was cloned upstream of the luciferase reporter. MDA‐MB‐231 cells were co‐transfected with the reporter plasmid and SMAD3 expression plasmid. Luciferase activity was measured 24–48 h after transfection using the Dual‐Luciferase Reporter Assay System (Novazan, China), and normalized to Renilla luciferase activity.

### 5.16 Quantitative Methylation‐Specific PCR

Genomic DNA was isolated from formalin‐fixed paraffin‐embedded (FFPE) TNBC tissues (*n* = 79) using the QIAamp DNA FFPE Tissue Kit (Qiagen, Germany) following the manufacturer's instructions. Prior to methylation analysis, DNA underwent bisulfite conversion. TNFRSF19 promoter methylation was quantified by quantitative methylation‐specific PCR (qMSP) using EpiTect MethyLight Master Mix (Qiagen) with specific primers and a methylation‐specific probe. ACTB served as an internal reference for normalization. Relative methylation levels were calculated using the 2^‐ΔCt method. Relative methylation levels were dichotomized into “high” and “low” groups based on the median 2^‐ΔCt value for correlation analyses. The primers and probes used in qMSP are listed in Table S3.

### 5.17 Seahorse Analysis

Oxygen consumption rate (OCR) was measured using a Seahorse XFe96 Analyzer (Agilent, USA). Cells were seeded in 96‐well plates and treated with doxorubicin. ATP production, maximal respiration, and spare capacity were determined following sequential injections of oligomycin, FCCP, and rotenone/antimycin A according to the manufacturer's protocol.

### 5.18 Mitochondrial Membrane Potential and Mitochondrial ROS Measurement

Mitochondrial membrane potential (Δψm) was assessed with JC‐1 staining (Abcam, USA), quantified as the red/green fluorescence ratio. Mitochondrial ROS was measured using MitoSOX Green (Invitrogen, USA) and analyzed by flow cytometry. These assays evaluated the effects of TNFRSF19 on mitochondrial quality control and oxidative stress under chemotherapeutic stress.

### 5.19 Statistical Analysis

Statistical analyses were conducted using R software and GraphPad Prism. Experiments were conducted at least three times unless stated otherwise. Data are presented as mean ± SD. Group comparisons were carried out using two‐way ANOVA, one‐way ANOVA, or two‐tailed unpaired Student's t‐test, unless indicated otherwise. Survival analyses were performed using the R package survminer. Kaplan–Meier curves were generated and compared using the log‐rank test. Statistical significance was defined as p < 0.05.

## Author Contributions

**Shiyang LiuChenguang Liu**: conceptualization, methodology, software, data curation, investigation, writing – original draft, formal analysis, visualization. **Chenguang Liu**: writing – review and editing, visualization, validation, methodology. **Weihong Zheng**: software, formal analysis, project administration, resources. **Xiaofei Tong**: investigation, validation, formal analysis. **Zhengwei Gui**: project administration, supervision. **Zonghong Lu**: formal analysis, validation. **Sun Ieong**: data curation, investigation. **Lin Zhang**: conceptualization, writing – review and editing, resources, supervision, project administration, methodology, funding acquisition.

## Funding

This research was funded by National Natural Science Foundation of China (Grant No 21834002).

## Conflicts of Interest

The authors declare no conflicts of interest.

## Supporting information




**Supporting File**: advs77529‐sup‐0001‐SuppMat.docx.

## Data Availability

All the data generated or analyzed during the study are included in this article or are available upon request.
