# Supplementary material for: Epigenetic Reactivation of TNFRSF19 Suppresses Mitophagy and Sensitizes Triple‐Negative Breast Cancer to Doxorubicin
Source: Adv Sci (Weinh). 2026 Aug 31:e77529. Online ahead of print. doi: 10.1002/advs.77529 (PMC13530348; doi:10.1002/advs.77529)
Supplement: Supplementary file 1 [file ADVS-9999-e77529-s001.docx]

**Table S1.** The Correlation between Clinical Pathological Features and TNFRSF19 Protein Expression

| **Clinical Pathological Features** | **TNFRSF19-High**  **n=41（%）** | **TNFRSF19-Low**  **n=38（%）** | ***P*** |
| --- | --- | --- | --- |
| **Age** |  |  | 0.4495648 |
| ≥ 45 | 14（34.15%） | 10（26.32%） |  |
| < 45 | 27（65.85%） | 28（73.68%） |  |
| **Tumor size** |  |  | 0.591302 |
| < 2 cm | 8（19.51%） | 8（21.05%） |  |
| 2-5 cm | 24（58.54%） | 25（65.79%） |  |
| ≥ 5 cm | 9（21.95%） | 5（13.16%） |  |
| **Lymph node metastasis status before neoadjuvant chemotherapy ^a^** |  |  | 0.4703814 |
| Yes | 26（63.41%） | 27（71.05%） |  |
| No | 15（36.59%） | 11（28.95%） |  |
| **Lymph node metastasis status after neoadjuvant chemotherapy ^b^** |  |  | 0.1454867 |
| Yes | 9（21.95%） | 14（36.84%） |  |
| No | 32（78.05%） | 24（63.16%） |  |
| **Histological grade** |  |  | 0.08173631 |
| I-II | 6（14.63%） | 14（36.84%） |  |
| III | 22（53.66%） | 19（50.00%） |  |
| NA | 13（31.71%） | 5（13.16%） |  |
| **Miller-Payne grade ^c^** |  |  | **0.00000414** |
| G1-G3 | 9（21.95%） | 28（73.68%） |  |
| G4-G5 | 32（78.05%） | 10（26.32%） |  |
| **Pathological complete response ^d^** |  |  | **0.004475** |
| Yes | 20（48.78%） | 7（18.42%） |  |
| No | 21（51.22%） | 31（81.58%） |  |

a. Lymph node metastasis before neoadjuvant chemotherapy refers to metastasis detected by imaging as potentially present in the axillary lymph nodes, and subsequently confirmed by fine-needle aspiration (FNA).

b. Lymph node metastasis after neoadjuvant chemotherapy refers to metastasis pathologically confirmed to be present in the axillary lymph nodes in the postoperative specimens.

c. Miller-Payne Grade G4-G5 indicates that, compared with the pre-neoadjuvant chemotherapy core needle biopsy tissue, the post-neoadjuvant chemotherapy surgical specimen tissue shows a reduction of tumor cells exceeding 90% or the absence of invasive carcinoma cells.

d. pCR (Pathological Complete Response) is defined as the absence of residual tumor in both the primary breast lesion and the axillary lymph nodes.

**Table S2.** Antibodies used in this study**.**

| Antibody | Species, company and catalogue number | Antibody dilution |
| --- | --- | --- |
| TNFRSF19 | Mouse, SantaCruz Biotechnology, #sc-398526 | 1:200 |
| PINK1 | Rabbit, Proteintech, #23274-1-AP | 1:500 |
| Parkin | Rabbit, Proteintech, #14060-1-AP | 1:500 |
| TOM20 | Rabbit, Proteintech, #11802-1-AP | 1:2000 |
| LC3B | Rabbit, CST, #2775 | 1:50 (IHC) |
| p62 | Rabbit, Proteintech, #18420-1-AP | 1:5000 |
| γH2AX | Rabbit, Abcam, #ab81299 | 1:2000 |
| Cleaved-caspase3 | Rabbit, HUABIO, #ET1602-47 | 1:500 |
| TGFBR1 | Rabbit, affinity, #AF5347 | 1:500 |
| SMAD2 | Rabbit, abcam, #ab40855 | 1:2000 |
| SMAD3 | Rabbit, abclonal, #A19115 | 1:2000 |
| SMAD4 | Rabbit, Proteintech, #10231-1-AP | 1:500 |
| SMAD2/3 | Rabbit, affinity, #AF6367 | 1:500 |
| p-SMAD2 | Rabbit, affinity, #AF8314 | 1:500 |
| p-SMAD3 | Rabbit, affinity, #AF8315 | 1:1000 |
| GAPDH | Rabbit, Proteintech, #60004-1-Ig | 1:4000 |
| GRP78 | Rabbit, proteintech, #11587-1-AP | 1:2000 |
| IRE1 | Rabbit, proteintech, #27528-1-AP | 1:500 |
| PERK | Rabbit, proteintech, #24390-1-AP | 1:500 |
| CHOP | Signalway antibody, #40744 | 1:500 |
| MEK1/2 | Rabbit, HUABIO, #ET1602-3 | 1:1000 |
| ERK1/2 | Rabbit, HUABIO, #ET1601-29 | 1:5000 |
| p-MEK1/2 | Rabbit, Affinity, #AF3385 | 1:500 |
| p-ERK1/2 | Rabbit, HUABIO, #ET1610-13 | 1:500 |

**Table S3. List of primers sequences used in quantitative methylation-specific PCR**

| **Gene** | **primer** | **5’→3’sequences** | **Application** | **Size**  **(bp)** | **Tm (**$\mathbf{℃}$**)** |
| --- | --- | --- | --- | --- | --- |
| *BACTIN* | Forward  Reverse  Probe | TGGTGATGGAGGAGGTTTAGTAAGT  AACCAATAAAACCTACTCCTCCCTTAA  ACCACCACCCAACACACAATAACAAACACA | MSP-M | 132 | 60 |
| *TNFRSF19* | Forward  Reverse  Probe | CGTTGCGAGGGTGCGGTC  CCCGAATTCGCCTCCTAC  TCGTTTCGGGGCGAGTGTTTTTCGG | MSP-M | 76 | 60 |

**Supplementary Figures**

**Figure S1. TNFRSF19 overexpression suppresses cell viability, while knockdown exerts no significant effect under basal culture conditions.** (A-B) CCK-8 assays measuring the viability of TNBC cells transfected with pcDNA (control) or OE-TNFRSF19 plasmids at 24, 48, 72, and 96 h. (C-D) CCK-8 assays measuring the viability of TNBC cells transfected with shNC (control) or sh-TNFRSF19 plasmids at the indicated time points. Data are shown as mean ± SD. Statistical significance was determined by two-way ANOVA followed by multiple comparisons test. ***P < 0.001; ****P < 0.0001; ns, not significant.

**Supplementary Figures**

**Figure S2. TNFRSF19 knockdown promotes mitophagy in TNBC cells.** ‌(A)‌ Western blot analysis of PINK1, Parkin, TOM20, and p62 in TNFRSF19-knockdown TNBC cells. ‌(B)‌ Western blot analysis demonstrates that the mitophagy inhibitor Mdivi-1 increases γH2AX and cleaved caspase-3 expression in TNFRSF19-knockdown cells treated with doxorubicin.

**Supplementary Figures**

**Figure S3. TNFRSF19 suppresses mitophagy initiation as assessed by LC3-II turnover analysis in the presence of Bafilomycin A1.** (A) Representative Western blot images showing the protein levels of LC3B-I/II, p62, TOM20, PINK1, and Parkin in MDA-MB-231 and BT-549 cells transfected with pcDNA or TNFRSF19 overexpression plasmid (OE-TNFRSF19), and treated with or without Bafilomycin A1 (BafA1, 100 nM) for 24 h. (B–F) Quantification of LC3-II, p62, TOM20, PINK1, and Parkin normalized to GAPDH. Data are presented as mean ± SD from three independent experiments (n = 3). Statistical significance was determined by one-way ANOVA followed by Tukey’s post hoc test. *P < 0.05, **P < 0.01, ***P < 0.001, ****P < 0.0001.

**Supplementary Figures**

**Figure S4. TNFRSF19 enhances doxorubicin sensitivity independently of MAPK pathway.** ‌(A)‌ Flow cytometry analysis shows that the MAPK pathway inhibitor U0126 failed to reverse the increased apoptosis rates enhanced by TNFRSF19 overexpression under doxorubicin treatment. ‌(B)‌ Western blot shows U0126 failed to reverse the increased γH2AX and cleaved caspase-3 expression in TNBC cells with TNFRSF19 overexpression combined with doxorubicin treatment. Student’s t-test was used for analysis (* P<0.05, ** P<0.01). ns, not significance.

**Supplementary Figures**

**Figure S5. TNFRSF19-regulated mitophagy is independent of MAPK pathway-mediated endoplasmic reticulum stress, and doxorubicin treatment does not alter the paraptosis-associated marker ALIX.** ‌(A)‌ Western blot shows the MEK1/2 inhibitor U0126 and the ERS inhibitor 4-PBA failed to counteract the TNFRSF19-mediated mitophagy inhibition. ‌(B)‌ Western blot shows the mitophagy activator CCCP did not affect the TNFRSF19-driven activation of MAPK pathway and ERS. ‌(C)‌ Western blot analysis showing that doxorubicin treatment did not alter the expression of the paraptosis-associated marker ALIX in control or TNFRSF19-overexpressing cells.

**Supplementary Figures**

**Figure S6. TNFRSF19 inhibits the formation of the Smad2-Smad4 complex in TNBC cells.** ‌(A)‌ GSEA analysis of TCGA-TNBC data show that TGF-β related pathway was negatively correlated with TNFRSF19 expression. ‌(B)‌ His-tag immunoprecipitation after co-expression of HA-SMAD4 with His-SMAD2 or TNFRSF19, followed by immunoblotting with anti-His and anti-HA antibodies. ‌(C) Anti-Smad2 immunoprecipitation assay evaluating endogenous Smad2/4 interactions in wild-type versus TNFRSF19-knockdown BT-549 cells, with or without TGFβ stimulation (5 ng/mL).

**Supplementary Figures**

**Figure S7. Validation of TNFRSF19-mediated regulation of SMAD2/3 phosphorylation in TNBC cells.** (A) Western blot analysis demonstrating that TNFRSF19 knockdown in MDA-MB-231 cells increased SMAD2/3 phosphorylation levels. (B) Western blot analysis showing that TNFRSF19 overexpression in BT-549 cells reduced SMAD2/3 phosphorylation levels.

**Supplementary Figures**

**Figure S8. Correlation of PINK1 with SMAD2/3 in TCGA-TNBC.**

(A) Spearman correlation showing a positive correlation between PINK1 and SMAD2 mRNA expression in the TCGA-TNBC cohort (R = 0.199, P = 0.014). (B) Spearman correlation showing a positive correlation between PINK1 and SMAD3 mRNA expression in the TCGA-TNBC cohort (R = 0.405, P < 0.001).

**Supplementary Figures**

**Figure S9. SMAD3 overexpression reverses TNFRSF19-mediated enhancement of doxorubicin-induced DNA damage in TNBC cells.** Western blot analysis of γH2AX in MDA-MB-231 and BT-549 cells. Cells were treated with doxorubicin (DOX) and transfected with TNFRSF19 overexpression plasmid (OE-TNFRSF19) alone or co-transfected with OE-TNFRSF19 and SMAD3 overexpression plasmid (OE-SMAD3).

**Supplementary Figures**

**Figure S10. The intracellular domain of TNFRSF19 is required for the regulation of PINK1/Parkin-mediated mitophagy.** Western blot analysis of PINK1, Parkin, p62, and TOM20 expression in MDA-MB-231 and BT-549 cells transfected with vector control, full-length TNFRSF19, or TNFRSF19-ΔICD mutant. Compared with full-length TNFRSF19, the TNFRSF19-ΔICD mutant failed to induce the changes associated with mitophagy inhibition. **Supplementary Figures**

**Figure S11. TNFRSF19 promoter hypermethylation is associated with reduced protein expression and poor chemotherapy response in TNBC.**

(A) TNFRSF19 promoter methylation was compared between tumors with high and low TNFRSF19 protein expression in an institutional TNBC cohort (n = 79). Tumors with low TNFRSF19 protein levels showed significantly higher promoter methylation. (B) TNFRSF19 promoter methylation was compared between patients achieving pathological complete response (pCR) and those who did not achieve pCR (no-pCR). Patients with no-pCR exhibited significantly higher methylation levels. (C) TNFRSF19 promoter methylation was compared across different Miller–Payne grades (G1–G3 versus G4–G5), showing that patients with higher-grade responses (G4–G5) exhibit lower methylation levels. Data are presented as boxplots and normalized to ACTB. Statistical significance was determined using Mann–Whitney test. *P < 0.05, **P < 0.01, ***P < 0.001, ****P < 0.0001.
